# *GIGANTEA* influences leaf senescence in trees in two different ways

**DOI:** 10.1093/plphys/kiab439

**Published:** 2021-09-23

**Authors:** Nazeer Fataftah, Pushan Bag, Domenique André, Jenna Lihavainen, Bo Zhang, Pär K Ingvarsson, Ove Nilsson, Stefan Jansson

**Affiliations:** 1 Department of Plant Physiology, Umeå Plant Science Centre, Umeå University, Umeå, Sweden; 2 Department of Forest Genetics and Plant Physiology, Umeå Plant Science Centre, Swedish University of Agricultural Sciences (SLU), Umeå, Sweden; 3 Department of Plant Biology, Swedish University of Agricultural Sciences, Uppsala, Sweden

## Abstract

*GIGANTEA* (*GI*) genes have a central role in plant development and influence several processes. Hybrid aspen T89 (*Populus tremula x tremuloides*) trees with low *GI* expression engineered through RNAi show severely compromised growth. To study the effect of reduced *GI* expression on leaf traits with special emphasis on leaf senescence, we grafted GI-RNAi scions onto wild-type rootstocks and successfully restored growth of the scions. The RNAi line had a distorted leaf shape and reduced photosynthesis, probably caused by modulation of phloem or stomatal function, increased starch accumulation, a higher carbon-to-nitrogen ratio, and reduced capacity to withstand moderate light stress. GI-RNAi also induced senescence under long day (LD) and moderate light conditions. Furthermore, the GI-RNAi lines were affected in their capacity to respond to “autumn environmental cues” inducing senescence, a type of leaf senescence that has physiological and biochemical characteristics that differ from those of senescence induced directly by stress under LD conditions. Overexpression of *GI* delayed senescence under simulated autumn conditions. The two different effects on leaf senescence under LD or simulated autumn conditions were not affected by the expression of *FLOWERING LOCUS T*. *GI* expression regulated leaf senescence locally—the phenotype followed the genotype of the branch, independent of its position on the tree—and trees with modified gene expression were affected in a similar way when grown in the field as under controlled conditions. Taken together, GI plays a central role in sensing environmental changes during autumn and determining the appropriate timing for leaf senescence in *Populus*.

## Teaser

In the autumn deciduous trees respond to environmental signals and induce leaf senescence, and two different senescence pathways can be distinguished

## Introduction

Every year deciduous trees go through a seasonal cycle of bud flush, growth, vegetative growth cessation, leaf senescence, dormancy, and development of cold hardiness. This cycle is important for survival during the boreal winter and has a large effect on overall energy and nutrient balance. To properly respond to seasonal cues, trees have developed a complex regulatory network that integrates internal and external factors such as age, photoperiod, temperature, and nutrient status to set the appropriate time at which to switch from a stage of growth and photosynthesis to a stage of survival and nutrient remobilization. Obviously, there is a tradeoff between growth and survival/remobilization, causing evolutionary pressure to optimize the timing of phenological events, and this has resulted in considerable local adaptation of aspen (*Populus tremula*) phenology traits such as bud set, which can also be observed in, for example, genome-wide association studies (Wang et al., 2018).

In comparison to growth arrest and bud set, the regulation of autumn leaf senescence in trees is less well understood. At least in aspen, a tree in a given environment initiates the senescence process on almost the same day every year (e.g. Keskitalo et al., 2005)—that is, it is under strict seasonal control—however, unlike bud set, photoperiod seems not to be the immediate, or only, trigger for senescence (Michelson et al., 2017). Temperature also affects autumn senescence in aspen (Fracheboud et al., 2009; Wu et al., 2018), but its effect is complex and probably less important than light for initiating autumn senescence under natural conditions. Accumulation of photosynthates (Lihavainen et al., 2020) and the levels of different nitrogen (N) species can also affect aspen leaf senescence, and severe drought or pathogen attack can, of course, initiate senescence at any time. Disentangling the different internal and external factors influencing senescence at the level of an individual tree has turned out to be a challenging task, not to mention understanding changes at the landscape level (Zani et al., 2020). However, understanding the internal and external factors influencing autumn senescence is key to predicting how climate change will modulate senescence in different tree species, and hence modeling the effects of climate change.

The “critical day length” regulates different aspects of plant growth, for example, flowering and vegetative growth (Andres and Coupland 2012; Pin and Nilsson 2012). The components of photo-periodically controlled flowering in annuals are well described, whereas our knowledge of photoperiodic control in trees is more fragmented although the same components appear to be involved. Growth cessation and bud set in *Populus* are induced by the shortening photoperiod in the autumn and are dependent on light input via phytochrome (PHY) and the circadian clock components LATE ELONGATED HYPOCOTYL 1; 2 and TIMING OF CAB EXPRESSION 1 via the CONSTANS/FLOWERING LOCUS T (FT) module (Olsen et al., 1997; Böhlenius et al., 2006; Ibáñez et al., 2010; Ding et al., 2018; Miskolczi et al., 2019). GIGANTEA (GI) and GI-like (GIL) proteins control, in interaction with FLAVIN-BINDING, KELCH REPEAT, F-BOX 1, and CYCLING DOF FACTOR 1, seasonal growth cessation in *Populus* through regulation of the *FT2* gene (Ding et al., 2018). However, GI is involved in many other aspects of plant physiology: drought tolerance, miRNA processing, chlorophyll accumulation, cold tolerance, salt tolerance, herbicide resistance, phloem function, and starch accumulation (Mishra and Panigrahi, 2015). Some of these effects may be consequences of the influence of changes in GI levels on the circadian clock and phenology, and separating primary from secondary effects is not an easy task. To study whether *GI* expression affects photosynthesis and leaf senescence in aspen, we set up a series of experiments using transgenic lines with modified expression of photoperiodic components in *Populus*, in particular grafted to one another*.* We found that GI is involved in the modulation of leaf physiology (e.g. gas exchange and the carbon-to-N [C/N] ratio) and that lowering *GI* expression could induce leaf senescence by photooxidative stress under moderate light, but also that *GI* expression modulates senescence in response to short days (SDs) and cold nights, clearly through a second physiological and molecular pathway. We use these findings to draw conclusions about the interaction between physiological traits mediated by *GI* expression, and leaf senescence, and how senescence is modulated at the whole tree level.

## Results

### Poor growth of GI-RNAi trees could be rescued by grafting scions onto wild-type rootstocks

**Figure 1:**
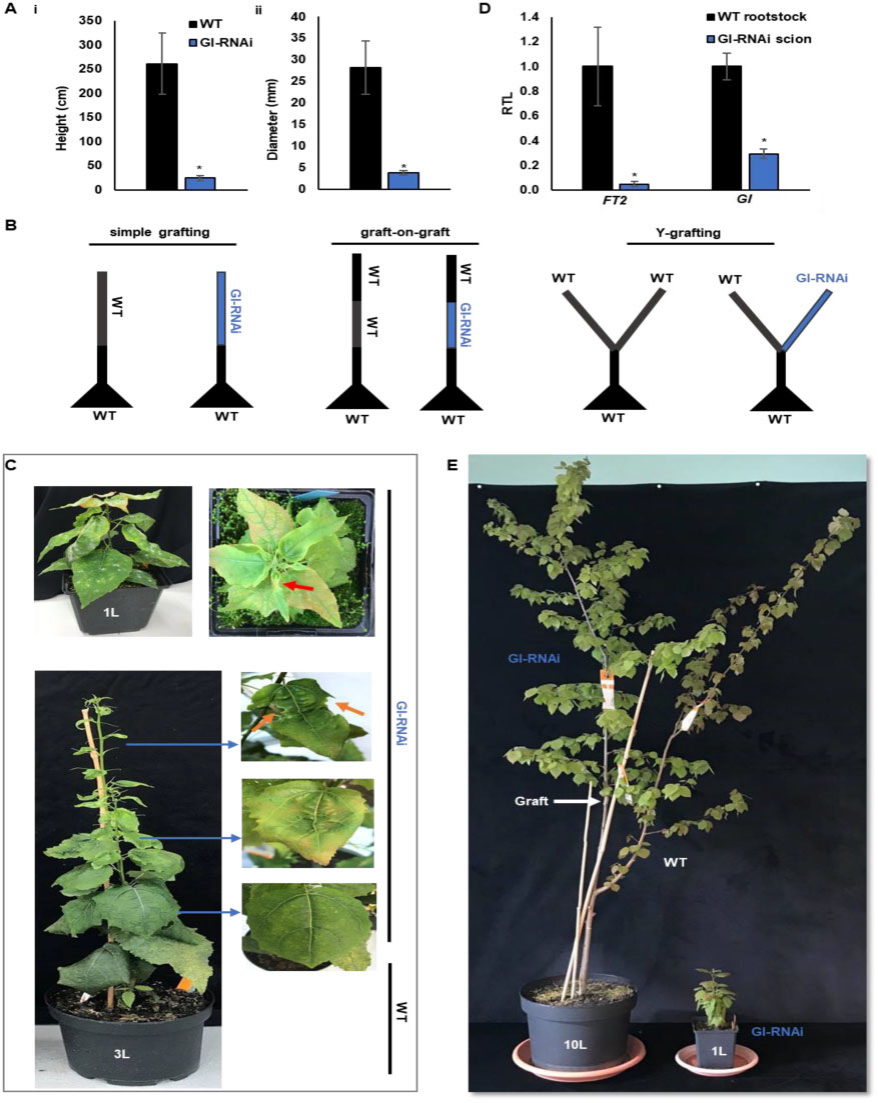
Rescuing the growth of GI-RNAi (line 8-2) by grafting onto WT rootstock. A, the growth of 3-year-old GI-RNAi trees in the field; (i–ii) show the height and stem diameter respectively; the bar is the average of nine tree values ± sd. B, illustrations show the different grafting methods. C, upper pictures show ungrafted GI-RNAi phenotype; the red arrow points to the most affected area of the leaf; the lower pictures show the phenotype of grafted GI-RNAi scion on WT rootstock and the leaf shape on the right; the orange arrow points toward a necrotic part (black region). D, expression of *GI and FT2* in the GI-RNAi scions and WT rootstock of grafted trees under LD (LD^18^) conditions; RTL, relative transcription level; the bar is the average of three biological replicates ± sd. E, the grafted GI-RNAi scion on WT rootstock in the third growth cycle. The asterisk represents a significant difference using *t* test; *P* < 0.05.

**Figure 2:**
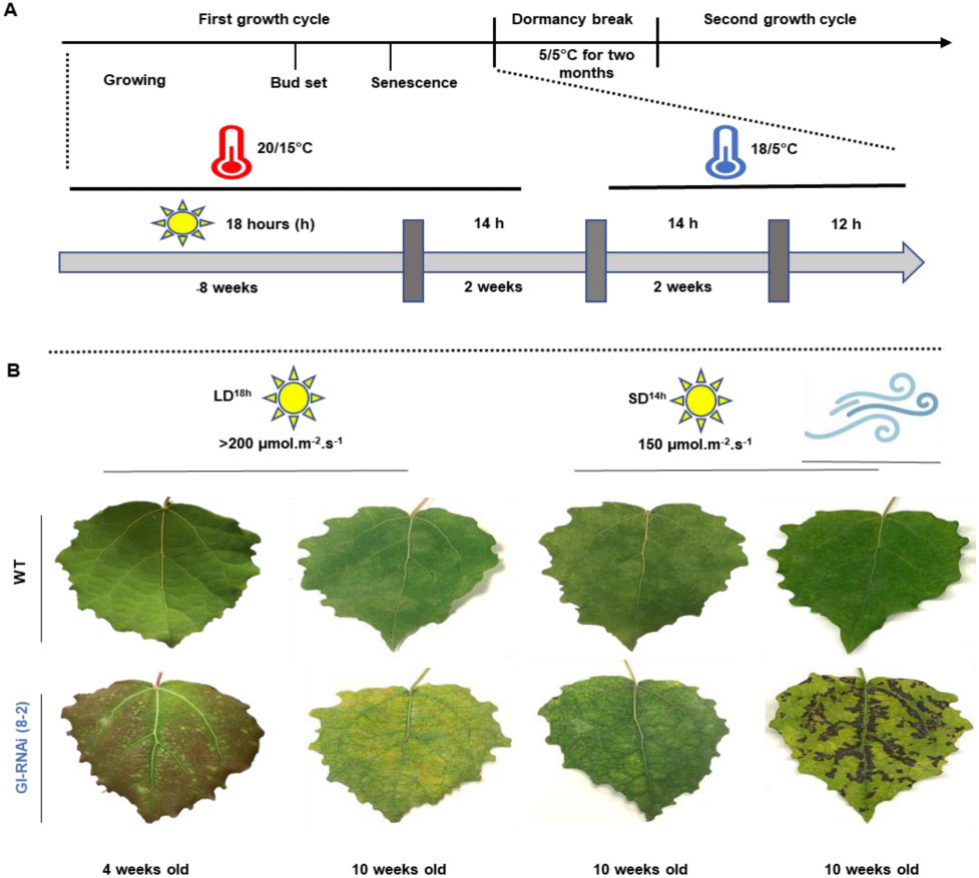
The leaves of grafted GI-RNAi (line 8-2) are sensitive to moderate light intensity. A, scheme of the different growth cycles and the indoor experimental setup simulating autumn conditions. B, the leaf phenotypes of trees in their second growth cycle under differing photoperiods and light intensities; the leaves to the right were transferred from LD^18^ ^h^ to SD^14^ ^h^ and lower light intensity; the right most two images show the necrosis/cell death caused by transpiration in GI-RNAi leaves after 1 week under enhanced air circulation; the upper bar represents the growth conditions; the images of the leaves were digitally extracted for comparison.

GI-RNAi lines have previously been shown to have severely reduced growth under climate chamber conditions (Ding et al., 2018). We selected one of the lines (8-2) previously studied and used this line for most of our studies; however, we also included a weaker RNAi line (line 1-1a) and a line overexpressing *GI* (*GI*-ox), see below. We grew the GI-RNAi (line 8-2) trees in the field (in southern Sweden), under natural conditions and found huge (ca. 10-fold) differences in growth (both stem height and diameter) between 3-year-old GI-RNAi (8-2) and wild-type (WT) trees (Figure 1A). The dramatic decrease in the growth and the early bud set of the GI-RNAi trees, both under controlled conditions and in the field, complicates studies of the effect of *GI* expression on tree physiology. However, given that GI regulates phenology through FT2, which is a mobile signal in *Populus* (Miskolczi et al., 2019), we tested whether grafting GI-RNAi (line 8-2) scions onto a WT rootstock (Figure 1B, “simple grafting”; GI-RNAi scion/WT rootstock), which allows mobile signals such as FT to move from WT leaves in the rootstock to the apex, could rescue the effects on growth and bud set. Self-grafted WT trees (WT scion/WT rootstock) were included as a control, and the grafted trees were grown under long-day (18-h light; LD^18^ ^h^) conditions. Growth of the GI-RNAi (8-2) scion was efficiently rescued by grafting onto a WT rootstock, and there was a striking difference between un-grafted and grafted GI-RNAi (8-2) scions (Figure 1C). The GI-RNAi (8-2) scions expressed the *GI* gene to ca. 25% of the levels in WT leaves on the rootstocks, and the mRNA levels from the *FT2* gene were low in GI-RNAi (8-2) scions (Figure 1D). Hence, the rescue of growth is likely to be caused by the movement of FT and/or other mobile signals like gibberellins from the rootstock to the scion. The rescuing of GI-RNAi (8-2) line growth was even more obvious in the second or third growth cycle after bud set/dormancy break (Supplemental Figure S1A; Figure 1E); a scheme representing the different growth cycles is shown in Figure 2A. Grafting made it possible to study the effect of *GI* expression on several aspects of development and physiology such as leaf morphology and senescence.

While grafting could rescue the bud set and growth phenotypes, leaf shape was still affected in GI-RNAi (8-2) scions. The first leaves that were established in GI-RNAi (8-2) scions after grafting had a normal shape but as the tree produced more leaves, they became increasingly aberrant with asymmetric growth around the main and secondary veins, and leaves at the top of the shoot showed symptoms of necrosis/cell death (Figure 1C). Accordingly, actively growing leaves of GI-RNAi (line 8-2) were less red, with lower anthocyanin and flavonol content than in the WT, even when they were grafted on the same WT rootstock using the “Y grafting method” (Figure 1B;Supplemental Figure S1, A–D). Similar leaf shape patterns were also noted in the un-grafted GI-RNAi (8-2) trees (Figure 1C). In the second growth cycle, when the buds were flushed after dormancy, leaves emerging from the seasonal buds had normal shapes, but as the shoot grew, subsequent leaves showed increasingly abnormal shapes indicating that the mobile signals from other parts of the tree that affected this phenotype were diluted as the tree grew.

### GI-RNAi leaves were sensitive to light and had lower CO_2_ assimilation rates

To study various aspects of leaf senescence in lines with modified *GI* expression, we studied the leaf physiology of two RNAi and one overexpression *GI* lines grown in several controlled environments. GI-RNAi (line 8-2) leaves were sensitive to light when the grafted trees were grown under LD^18^ ^h^ and standard light intensity in the greenhouse (200 µmol m^−2^. s^−1^) causing chlorosis of GI-RNAi (8-2) leaves (Figure 2B), indicative of photooxidative stress. Furthermore, the exposed leaves showed more red coloration (anthocyanins) between the veins (Figure 2B). However, unlike the growing leaf, in which anthocyanin is produced by default to prevent stresses, the anthocyanin in the RNAi (8-2) mature leaf is a consequence of light stress. This photooxidative phenotype could be recovered (“regreening”) when the trees were moved to SD (14-h light; SD^14^ ^h^) and lower light intensity (150 µmol m^−2^. s^−1^) conditions (Figure 2, A and B). In this context, it has been proposed that GI influences stomatal conductance in *Arabidopsis thaliana* (Ando et al., 2013), and Edwards et al. (2010) showed that GI functions in veins influencing the wall ingrowth of phloem parenchyma cells. It is likely that functional synchronization of stomata and veins could cause the above-mentioned phenotypes of GI-RNAi (8-2) leaves, for example, green parts under LD^18^ ^h^ and asymmetric growth close to the veins (Figures 1, C and 2, B). We shifted grafted trees in their second growth cycle from LD^18^ ^h^ to a growth chamber with SD^14^ ^h^ conditions, intending to simulate autumn conditions (Figure 2A). On multiple occasions, we noted necrosis in GI-RNAi (8-2) leaves by 1 week after the shift, whereas no such symptoms were found in the WT leaves (Figure 2B). Necrosis was more pronounced around the main and secondary veins, suggesting a connection with vascular tissue malfunction in GI-RNAi (8-2) leaves (Figure 2B). On the other hand, as the necrotic phenotype could possibly be associated with increased air circulation—we noted that leaves facing the fan suffered more from necrosis than the less exposed leaves on the other side of the trees—it is also feasible that stomatal malfunction may contribute to this phenotype.

**Figure 3:**
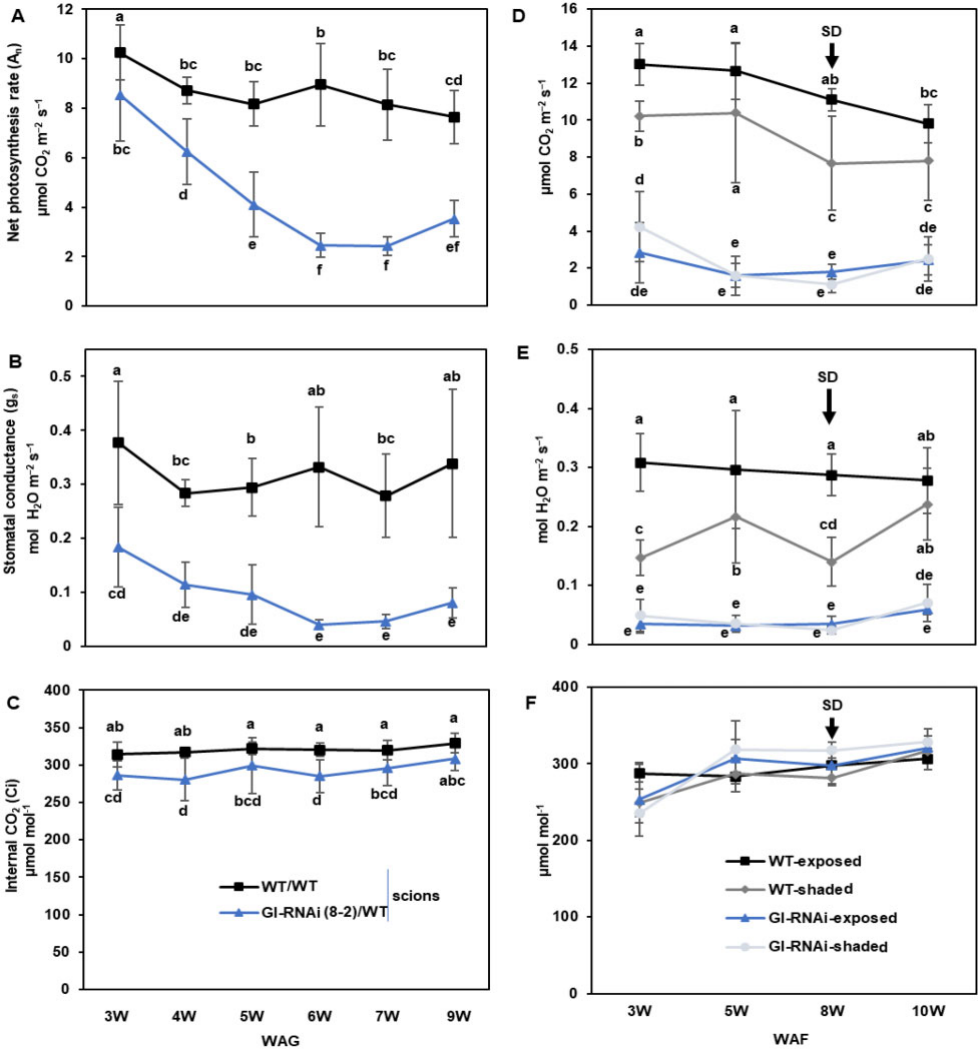
Gas exchange parameters of grafted WT and GI-RNAi (line 8-2) scions. A–C, A_n_, g_s_, and Ci respectively, for simple graft GI-RNAi or WT scions on WT rootstock under LD^18^ ^h^. D–F, A_n_, g_s_, and Ci respectively, for graft-on-graft trees that have WT scions on the top of grafted GI-RNAi; from 8 weeks after flushing (WAF) the trees were subjected to SD^14^ ^h^. Data points are the average of 4–6 scion values ± sd. Different letters represent significant differences using analysis of variance analysis; *P* < 0.05.

These observations indicated that photosynthetic capacity may be compromised in GI-RNAi (8-2) leaves when incident light intensity exceeds the capacity to utilize the light energy under conditions of low photosynthesis, causing photooxidative stress. However, the response was complex, as there were differences between the regions close to the veins and interveinal regions (compare Figures 1, C and 2, B). This prompted us to perform a detailed analysis of the photosynthetic performance of leaves of WT and GI-RNAi (8-2) grafted plants. Trees were kept under LD^18^ ^h^ conditions and the net CO_2_ assimilation rate (A_n_), stomatal conductance (g_s_), and the internal CO_2_ of the leaf (Ci) were measured weekly after the grafting event using an LI-6400XT portable photosynthesis system (LI-COR environmental company) instrument. The same set of leaves—the first established after grafting—was measured throughout the experiment. Three weeks after grafting, WT and GI-RNAi (8-2) leaves had relatively similar A_n_ (Figure 3A). However, A_n_ decreased rapidly with time in GI-RNAi scions and after 6 weeks it was only ca. 20% of that in WT scions (Figure 3A). g_s_ was already lower in GI-RNAi (8-2) 3 weeks after grafting and it decreased even further with time (Figure 3B), but Ci did not differ much either between the lines or over time (Figure 3C). To further study the relationship between various *GI* expression levels and these photosynthetic parameters (A_n_, g_s_, and Ci), we analyzed a second GI-RNAi line (1-1a, grafted onto the WT rootstock), in which *GI* mRNA levels were only moderately decreased (Supplemental Figure S2), and a line *GI*-ox (Ding et al., 2018). Line 1-1a showed little or no altered growth phenotype, no abnormal leaf shapes, and no signs of photooxidative stress under the conditions employed here, but it formed buds after only 2 months under LD^18^ ^h^ conditions. These lines did not differ from the WT with respect to gas exchange parameters (Supplemental Figure S3); clearly, more severe depletion of *GI* RNA was required to noticeably compromise photosynthetic performance than to affect bud set.

### Intrinsic properties of GI-RNAi leaves do not depend on position on the tree

As GI modulates growth arrest, decreased CO_2_ assimilation in GI-RNAi (line 8-2) could potentially be the result of an alteration in sink demand. Moreover, to test whether mobile signals move upward from the RNAi parts, we grafted a WT scion onto the top of GI-RNAi (8-2) grafted on WT to create “graft-on-graft trees” (Figure 1B). In the subsequent growth cycle after SD/dormancy conditions, followed by bud flush under LD^18^ ^h^ (Figure 2A), trees continued growing like the control WT self-grafted trees. No difference in stem diameter of WT and GI-RNAi parts on the same tree was observed (Supplemental Figure S4).

**Figure 4:**
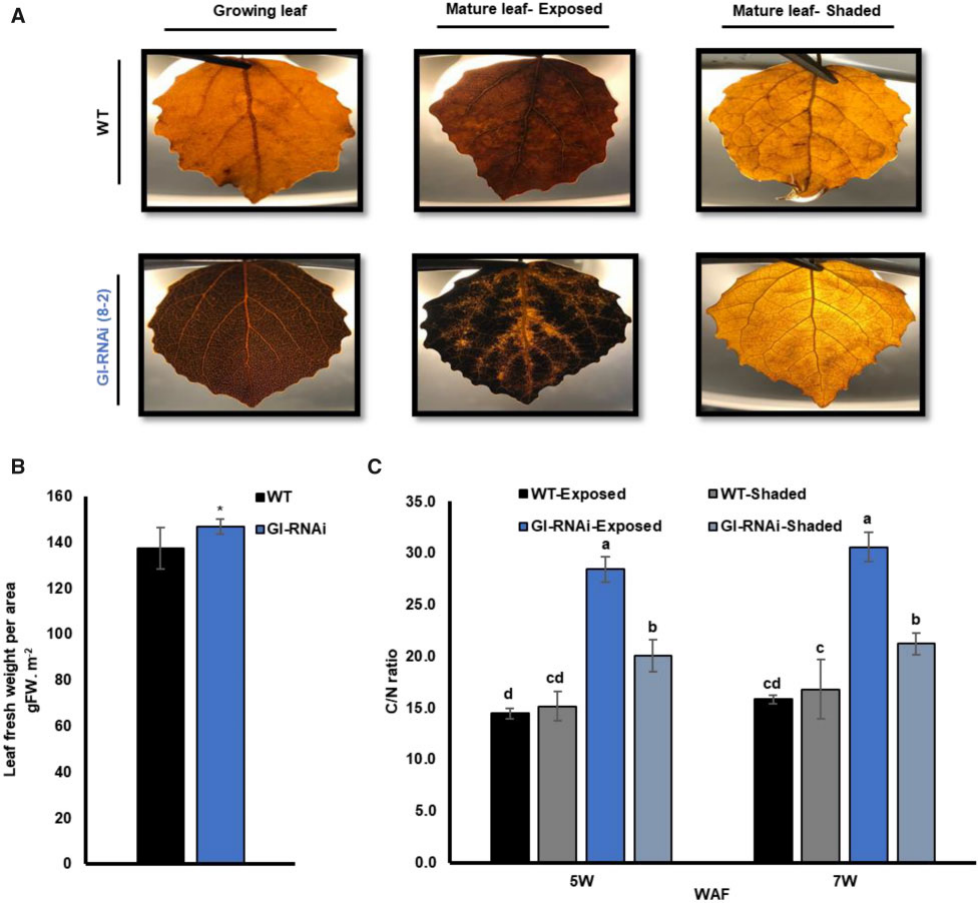
Starch levels and C/N ratios in WT and GI-RNAi (line 8-2) leaves on the same tree under LD^18^ ^h^. A, starch staining of exposed and shaded leaves from the top WT and GI-RNAi leaves on the graft-on-graft trees; the pictures were taken with illumination from the rear for improved visualization. B, fresh weight of leaves per unit area in the graft-on-graft trees; the bar is the average of 10 leaves ± sd. C, C/N ratio in the leaves of the graft-on-graft trees; the bar represents the average of four biological replicates from four tree values ± sd. The asterisk and different letters represent significant differences using a *t* test or analysis of variance, respectively; *P* < 0.05.

Here we also compared leaves that were shaded naturally by other leaves to study A_n_ and g_s_ of GI-RNAi (8-2) leaves under low light (˂100 µmol m^−2^. s^−1^), when leaves were not obviously under light stress. Shading decreased the A_n_ and g_s_ of the WT leaves compared to neighboring ones that were fully exposed to a light intensity of 200–300 µmol m^−2^. s^−1^ (Figure 3, D and E) but A_n_ and g_s_ were again much lower in both exposed and shaded GI-RNAi (8-2) leaves, under both LD^18^ ^h^ and SD^14^ ^h^ conditions (see Figure 2A for experimental setup), than in the WT leaves at the top of the same tree or self-grafted WT (Figure 3, D and E; Supplemental Figure S5, A and B). We also analyzed starch accumulation by these trees, and GI-RNAi (line 8-2) leaves contained much higher starch levels than WT leaves (both below and above the GI-RNAi scion); this was evident in both growing and mature leaves (Figure 4A;Supplemental Figure S4), particularly in exposed conditions; as expected, shaded leaves contained less starch and the difference between GI-RNAi and WT was also less pronounced. The starch accumulation was also reflected in an increase in fresh weight per unit area of the leaves by 7% (Figure 4B) and their C/N ratio was increased in exposed and shaded leaves, but more pronounced in the exposed leaves of GI-RNAi (8-2) leaves (Figure 4C). It should also be pointed out that starch accumulation in GI-RNAi (8-2) leaves was less uniform than in WT: little starch accumulated along main and secondary veins, consistent with a role for GI in the function of stomata and/or veins (Figure 4A). Taken together, GI-RNAi (line 8-2) leaves had a lowered A_n_ but higher C/N ratio and starch accumulation in exposed leaves, even when growing between two sections of WT stems. Thus, the effect is an intrinsic property of the GI-RNAi leaves, not of the sink/source activities. Furthermore, starch accumulation did not explain the lowering A_n_ and g_s_ in shaded leaves, indicating that stomatal, rather than phloem loading, malfunction was more important in explaining the lower A_n_.

**Figure 5:**
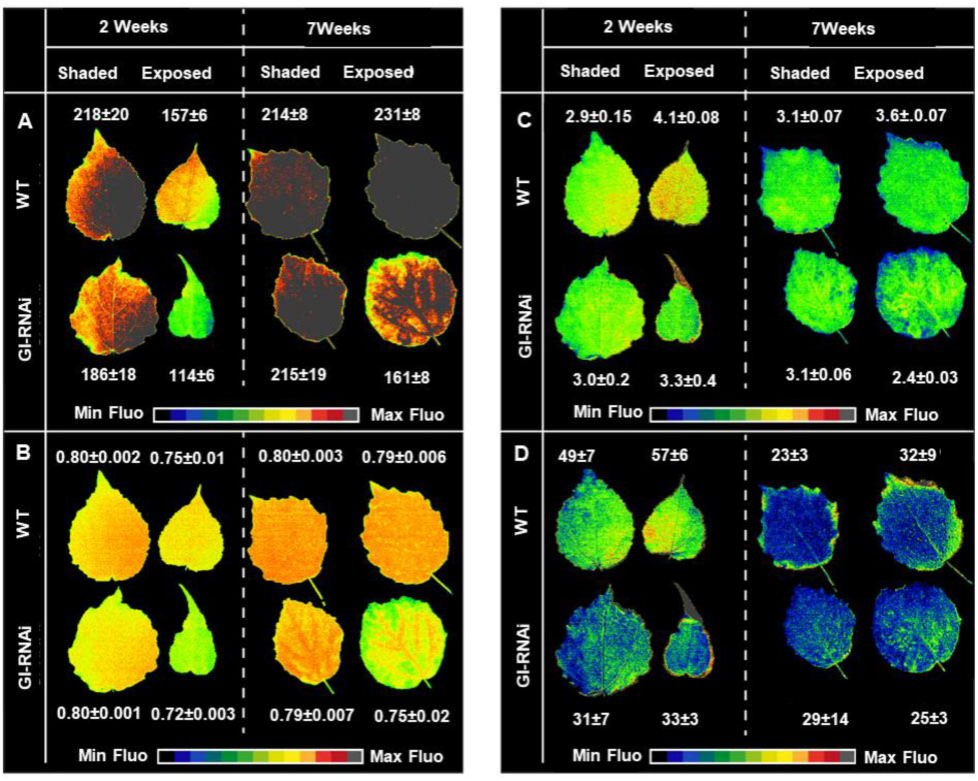
Photosynthetic performance of GI-RNAi (line 8-2) compared to WT at two different stages (2 and 7 weeks) after bud flush of graft-on-graft trees, for exposed or shaded leaves. A, Fm (scale 1–200 r.u.), B, maximum quantum yield of PSII (Fv/Fm, scale 0.01–0.80), and C, fast component of nonphotochemical energy dissipation (NPQ, scale 0.01–5) measured with saturating pulses (SPs) under 2,600 µmol constant actinic light. Representative images of fast and slow components of NPQ are shown for different SP applied during the measurement period. D, the slow component of NPQ measured from direct fluorescence from the last flash (14th) of the dark recovery period. The leaves were obtained from three independent grafted trees. The images of the leaves were digitally extracted for comparison.

The chlorophyll and starch staining data indicated that there were spatial differences in chlorophyll fluorescence properties within GI-RNAi (8-2) leaves. Analyzing chlorophyll fluorescence using the SPEEDZEN imaging system is a powerful method that can give information about different photosynthetic properties with spatial resolution, therefore we studied exposed and shaded leaves of WT and GI-RNAi (line 8-2) on the same graft-on-graft trees on 2 and 7 weeks after flushing (Figure 5), using SPEEDZEN imaging. In general, maximum fluorescence (Fm) was, overall, lower in GI-RNAi leaves (Figure 5A) indicating that photosystem II (PSII) was inhibited or quenched. Fv/Fm (maximum quantum yield of photosystem II) (Figure 5B) tended also to be lower, indicating that PSII activity was in some way reduced as a consequence of quenching or photoinhibition. Some differences could also be noted in the amounts of the fast (energy dependent quenching (qE); Figure 5C) and slow (zeaxanthin dependent quenching (qZ)/photoinhibitory quenching (qI), Figure 5D) components of nonphotochemical chlorophyll fluorescence quenching (NPQ), which describes the dissipation of excess absorbed light energy into heat (Horton et al., 1996; Lambrev et al., 2012; Bag et al., 2020). The lower amplitude of NPQ (fast + slow) in GI-RNAi line and their spatial distribution in the leaf could be caused by reduced Calvin cycle activity due to, for example, lowered activity of phloem transfer cells or stomatal malfunctions. Taken together, our data suggest that lowered *GI* expression primarily affects the photosynthetic dark reaction and that the effects on the light reaction are indirect.

### RNAi of *GI* affected leaf senescence by two different pathways

Using studies on the physiology and metabolism of senescing leaves of stem-girdled aspen trunks grown under natural conditions in the field along with nongirdled trunks on the same tree, we have recently been able to separate different patterns of leaf senescence in aspen. Girdling resulted in the early onset of senescence, overriding the “normal phenological control” of autumn senescence, that is, the fact that a given tree genotype induces senescence at approximately the same date every year (Lihavainen et al., 2020). However, as we saw more similarities, such as high C/N ratio and anthocyanin level, between stress-induced senescence in GI-RNAi (line 8-2) leaves growing in LD^18^ ^h^ and senescence in girdled aspens, rather than in aspens undergoing typical autumn senescence in the field, we set out to induce autumn senescence in a growth chamber by changing light conditions and temperature. We included both grafted trees from their first and second growth cycle and the “graft-on-grafted” trees in this experiment. In addition, we included trees grafted with the weaker GI-RNAi line (1-1a), *GI*-ox, and nongrafted trees of each genotype. To simulate autumn, the trees were moved from LD^18^ ^h^ to SD^14^ ^h^ and after 2 weeks night temperature was lowered (18/5°C day/night; T^18/5°C^) (the experimental setup is shown in Figure 2A), resembling late August/early September conditions in Umeå.

**Figure 6:**
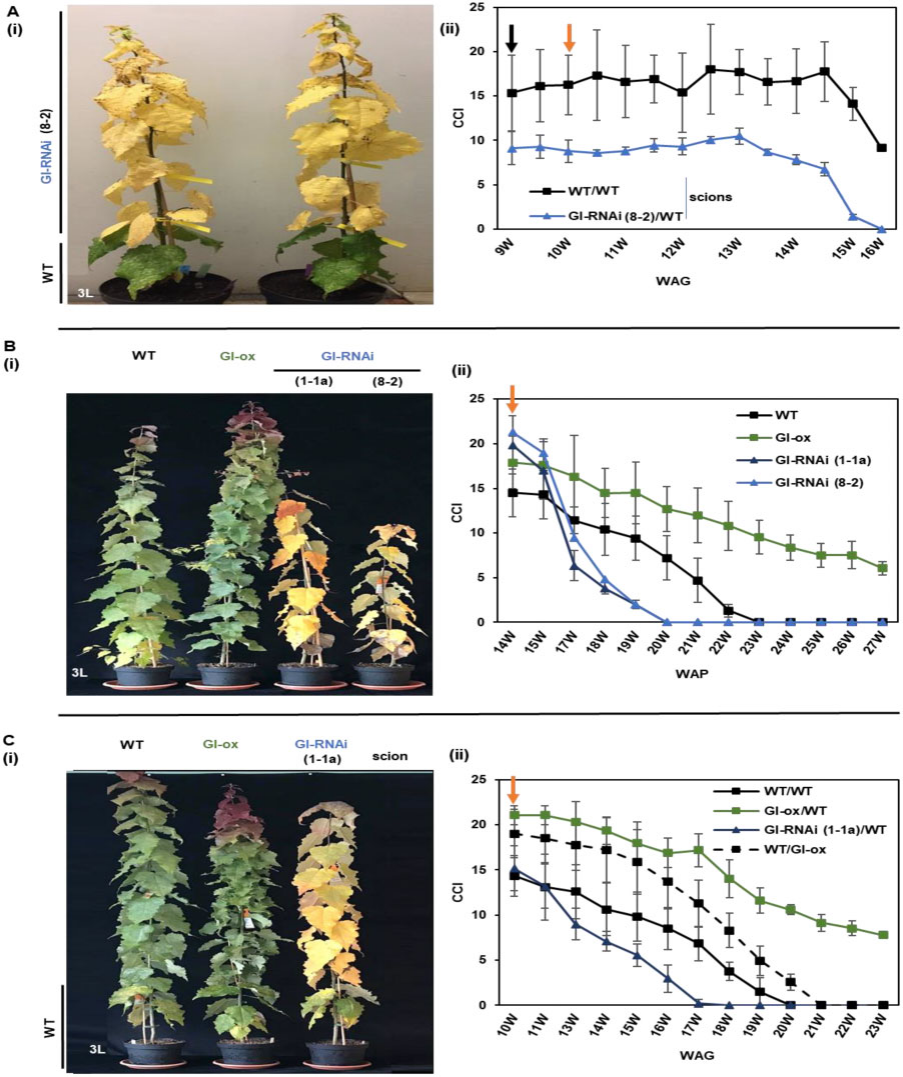
GI-RNAi leaves senesce earlier than WT leaves under simulated autumn conditions. A, senescence phenotypes of grafted GI-RNAi (line 8-2) and WT scions on a WT rootstock under simulated autumn conditions; the photograph of grafted GI-RNAi (line 8-2) on a WT rootstock (GI-RNAi (8-2)/WT) was taken at 15 weeks after grafting (WAG). B, the senescence phenotypes of nongrafted WT, *GI*-ox, and GI-RNAi (lines 1-1a and 8-2); the tree to the right is the only individual of the line 8-2 trees that grew more than 15 cm; the photo was taken at 20 weeks after potting. C, the senescence phenotypes of grafted WT, GI-ox, and GI-RNAi (line 1-1a) scions on a WT rootstock; the photograph was taken at 16 WAG. B(ii, CCI of nongrafted trees; (A, C(ii)) CCI of scions of grafted trees represented in the corresponding picture to the left. The data points are the averages of 4–6 tree values ± sd. The black arrow indicates when trees were subjected to short day condition, and the orange arrow represents when trees were subjected to cold nights.

Under these conditions, GI-RNAi (line 8-2) leaves always senesced earlier than WT leaves, as was evident visually or when the curve of chlorophyll content index (CCI) was compared between GI-RNAi (8-2) scion (GI-RNAi (8-2)/WT) and the scion of self-grafted WT (WT/WT) (Supplemental Figure 6, A (i)–(ii)). Under these simulated autumn conditions, the senescence phenotype was uniform in all leaves including the exposed and shaded leaves, and independent of the position on the tree (Figure 6A). GI-RNAi (8-2) leaves were typically shed 2 weeks before WT leaves. Furthermore, when ungrafted GI-RNAi line 1-1a trees were subjected to SD^14^ ^h^ T^18/5°C^, the leaves became senescent much earlier than the WT (Figure 6B), and the phenotype was reproduced when line 1-1a was grafted as scion or rootstock with WT (Figure 6C;Supplemental Figure S6, A and B). Obviously, although this line grew and photosynthesized like WT (with no sign of photooxidative stress), senescence induced by SD and lowering temperature was affected, so this senescence trait—autumn senescence—was more sensitive to decreased expression of *GI* than the other type of senescence caused by photooxidative stress and starch accumulation under LD^18^ ^h^ conditions, which hereafter we will term “premature senescence.” On the other hand, *GI*-ox delayed senescence by at least 6 weeks under SD^14^ ^h^ T^18/5°C^; both ungrafted and grafted branches displayed a delayed senescence phenotype (Figure 6, B and C; Supplemental Figure S6, C and D).

**Figure 7:**
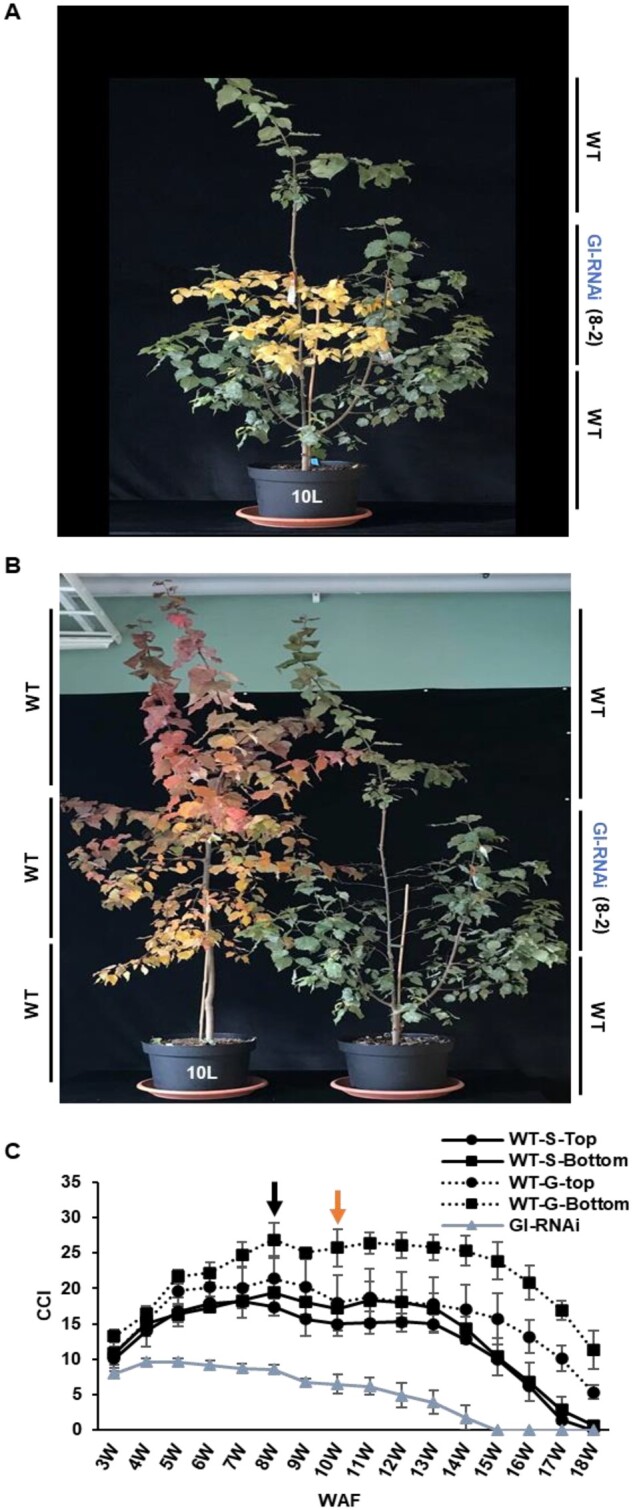
The senescence phenotype of graft-on-graft trees under simulated autumn conditions. A and B, the pictures represent the senescence phenotype in WT and GI-RNAi (line 8-2); the photographs were taken at, respectively, 14 and 16 WAF. C, CCI of leaves of graft-on-graft trees; WT-S: Self grafted WT. WT-G: WT scion grafted on GI-RNAi. The black arrow indicates when the trees were subjected to SDs, and the orange arrow shows when they were subjected to the cold night. The trees were grown in 10-L pots. The bar is the average of four tree values ± sd.

We also observed that GI-RNAi (line 8-2) leaves, which senesced earlier than WT, seemed to indirectly affect the senescence behavior of WT leaves; WT leaves that shared the same tree with GI-RNAi (8-2) leaves in the graft-on-graft plants had higher pre-senescence chlorophyll levels and entered senescence later than control grafted WT trees (Figure 7). We believe this to be a consequence of improved nutrient status; as GI-RNAi (8-2) leaves accumulate less N (and chlorophyll; Figures 4, C and 7), more N is available to WT leaves on the same tree during growth and this is even more the case when the GI-RNAi (8-2) leaves have started to senescence, and the remobilized mineral nutrients become available for the rest of the plant.

**Figure 8:**
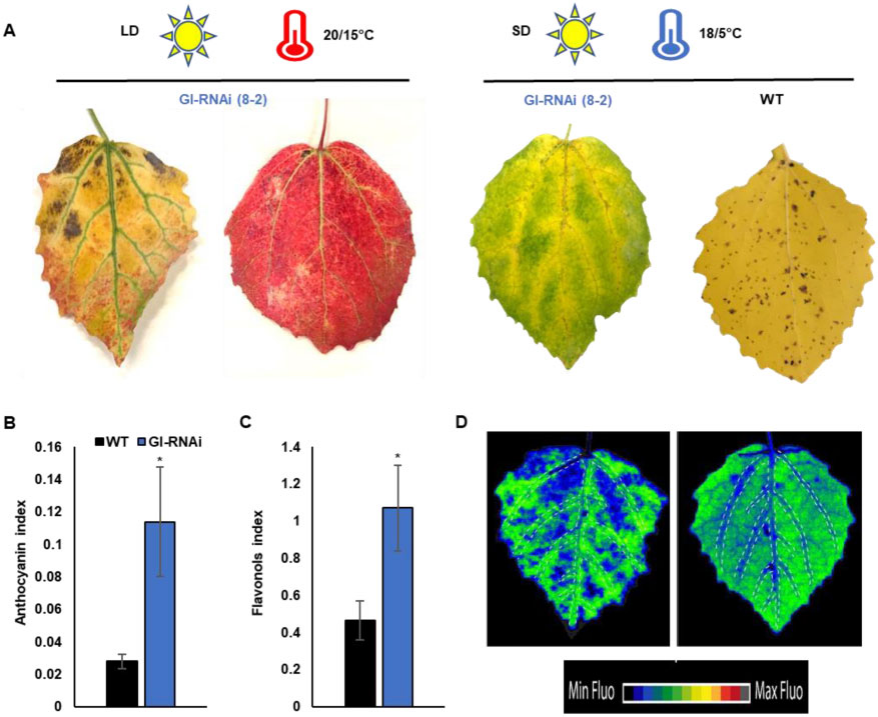
The two types of senescence in GI-RNAi (line 8-2) are dependent on environmental conditions. A, leaf senescence phenotype of GI-RNAi under LD^18^ ^h^ or SDs and cold nights; WT leaf represents typical uniform leaf senescence in the field and under simulated autumn conditions; the upper bar represents the growth conditions. B, anthocyanin index values for leaves under LD^18^ ^h^ conditions; C, flavonoid index values for leaves under LD^18^ ^h^ conditions. D, spatial patterns of chlorophyll fluorescence in GI-RNAi leaves under LD^18^ ^h^ (left leaf) and SDs and cold nights (right leaf); dashed lines indicate veins. The values in B and C are averages of four biological replicates ± sd. The asterisk represents a significant difference using a *t* test; *P* < 0.05. The images of the leaves were digitally extracted for comparison.

During the experiments when senescence was induced by SD and lowered temperature (SD^14^^–^^12^ ^h^ T^18/5°C^)—simulating autumn conditions—we noted differences in the patterns of leaf senescence in GI-RNAi leaves (in the stronger mutant line 8-2) compared to when senescence was induced under LD^18^ ^h^, “premature senescence.” “Autumn senescence” of GI-RNAi (8-2) was fairly uniform but it started along the main and secondary veins in both exposed and shaded leaves rather than interveinal regions (Figure 8A), while in some cases the veins stayed green similar to intervein than vein’s close regions. These regions were associated with previously mentioned phenotypes such as reduced starch accumulation or photooxidative stress in the exposed leaves (Figures 2 and 4, A). In WT and line 1-1a, senescence under “simulated autumn conditions” was more uniform (Figure 8A;Supplemental Figure S6B). In LD^18^ ^h^, “premature senescence” in GI-RNAi line 8-2 started in intervein regions, mainly in the exposed leaves. Under LD^18^ ^h^ conditions, leaves also produced more anthocyanin and flavonol compounds (Figure 8, B and C). The differences were obvious not only in the coloration of the leaves when visually inspected but also when the chlorophyll fluorescence patterns were compared (Figure 8D). Taken together, these observations show that reduced expression of *GI* in *Populus* leaves affected two different molecular or physiological pathways leading to leaf senescence, one of which made the strong RNAi line (8-2) leaves already more vulnerable to (photooxidative) stress during the stage of active growth of the tree and was associated with starch accumulation in the intervein regions. The second molecular pathway was induced by shortening the photoperiod and lowering the temperature, that is, resembling conditions that induce the autumn senescence that we study in the field. This type of autumn senescence was not associated with starch accumulation or photooxidative damage. We also subjected WT and GI-RNAi (line 8-2) leaves, grafted onto a WT rootstock, to a SPEEDZEN time-course analysis over 15 weeks (LD^18^ ^h^ to SD^14^ ^h^-to-SD^14^ ^h^ T^18/5°C^). The results (Supplemental Figure S7) were consistent with the lowering of GI expression both reducing the leaf’s ability to withstand photooxidative stress and changing senescence behavior when days became shorter and nights colder.

### *GI* expression also influences senescence under field conditions, whereas *FT* expression does not

In the laboratory, under LD^18^ ^h^ conditions, GI-RNAi trees were able to grow if provided with a mobile signal from a WT rootstock (i.e. when grafted on WT) but were still affected in the progression of leaf senescence. To see if the same was true under field conditions, we set up an outdoor experiment where trees were grown in pots but exposed to natural changes in light and temperature (in Umeå). Several different trees were used (with several replicates): (1) GI-RNAi (line 8-2) grafted onto WT and (2) control grafted WT, both in their second growth cycle. For these trees, WT buds on the rootstock were removed, producing trees with only one type of leaf after bud flush to check the senescence phenotype when the tree had only GI-RNAi (8-2) leaves. In addition, we included trees with GI-RNAi (8-2) and WT scions on the same WT rootstock, resulting in “Y grafting” (Figure 1B;Supplemental Figure S8B). In these trees, the WT scion in the Y graft grew faster and growth of GI-RNAi branches was suppressed under LD conditions, in contrast to the situation in simple grafted trees. Clearly, the WT scion was able to impose apical dominance over the GI-RNAi scion. After a cycle of dormancy/dormancy break, all trees were flushed in LD^18^ ^h^ T^20/15°C^ and moved outdoors in July (when the light period was ca. 20 h). The GI-RNAi parts of all trees set bud in July, while the WT in the two groups, including the trees that had GI-RNAi scions, did not form buds until October. GI-RNAi leaves senesced and were shed earlier than WT leaves (Supplemental Figure S8, C and D) and under these conditions senescence also started around the veins of GI-RNAi leaves in September (Supplemental Figure S8C). The effect of GI on aspen leaf senescence under field conditions appeared not to be mediated through FT. When FT-RNAi, WT, and overexpressing *FT* (*FT*-ox) aspen trees were grown under outdoor conditions, FT-RNAi set bud more than 1 month before the other lines, but senescence was not affected (Supplemental Figure S9A). This was also confirmed by measurements of senescence in 2-year-old trees of FT-RNAi, WT, and *FT*-ox in a field experiment in southern Sweden (Supplemental Figure S9B); again, in this case, bud set was affected in FT-RNAi trees but senescence was no different from WT.

## Discussion

In this contribution, we show that the level of *GI* expression affects autumn leaf senescence in aspen in a very complex way. We still have only a shallow understanding of autumn leaf senescence. Although it is a strictly regulated developmental program, much effort has been expended to identify genes regulating leaf senescence, how environmental conditions trigger the process, and which hormones are involved. There is little consensus on which genes, metabolites, or pathways provide the keys to the process. One obvious possibility is that if senescence is the default pathway for leaf development, the execution of which has to be prevented by a set of “blocking factors,” those that are removed could vary between conditions and species. Comparing genetically identical plant individuals experiencing different conditions is a useful way to understand complex phenomena, and has been widely used to understand senescence, although alternative approaches may give additional information. We have, for example, recently shown that different parts of the same tree can induce senescence in slightly different ways. Girdling one of the trunks in a multi-stem aspen individual introduced changes in leaf pigment content, metabolites, and senescence behavior; in other words, this treatment induced variation in different parts of a single individual. Here we explore a different strategy by studying senescence processes within a tree, in which parts of the tree have different genetic backgrounds exposed to the same environmental conditions. With this setup, we disentangle two modes of leaf senescence, expressed within the same tree. Reducing the expression of *GI* in controlled conditions was moderately stressful for the leaves under moderate light intensity and resulted in earlier senescence and anthocyanin accumulation, presumably because of fundamental changes in leaf physiology and photosynthetic characteristics. In Arabidopsis, lowered *GI* expression leads to malfunctioning phloem loading (Edwards et al., 2010), and our observations are consistent with compromised phloem export in *Populus* too. The early senescence we observed under LD conditions in GI-RNAi (the stronger line; 8-2) could therefore be analogous to the early senescence that can be induced by disrupted transport of photosynthates due to stem girdling (Lihavainen et al., 2020); lowered *GI* expression may disrupt transport out of the leaf. Another type of leaf senescence was observed when we exposed the trees to conditions that simulated autumn conditions: GI-RNAi leaves senesced earlier than WT leaves, but here the senescence process more resembled the “typical autumn senescence” that makes boreal forests colorful in a very consistent fashion every autumn. However, low *GI* expression also influenced the way in which the leaf experienced “autumn conditions” that initiate leaf senescence in a manner that we have studied extensively over the years in aspen (Bhalerao et al., 2003; Andersson et al., 2004; Keskitalo et al., 2005; Fracheboud et al., 2009; Edlund et al., 2017; Michelson et al., 2017); this is a process that, under natural conditions, is initiated on more or less the same date every year in a given genotype and is largely unaffected by weather. This type of senescence is quite different on a macroscopic level: leaves senesce in a more uniform way whether exposed or shaded leaves, and with less obvious signs of photooxidative damage. We believe that these two modes of leaf senescence are of wide physiological relevance but are hard to study under most conditions since they can occur in parallel, and our experimental manipulations—affecting the expression of *GI* or girdling experiments—have made it possible to better distinguish between the two modes. A clearer distinction between different aspects of leaf senescence would be useful for the community, and the mere fact that we are uncertain how to name them illustrates the lack of a conceptual framework. We decided to use the term “premature senescence” although “stress-induced senescence” would be an alternative name.

We also use our results to draw other conclusions about autumn senescence. First and foremost, although premature/stress-induced senescence clearly can be local, it has previously not been clear whether autumn senescence in a mature tree is local or systemic. Early observations that street lights may delay leaf fall (Matzke, 1936) have led to speculation that illumination of one part of the crown of a tree could keep that part green. The fact that this finding has not been possible to replicate, by others (e.g. Sarala et al., 2013) or ourselves (unpublished results), could be interpreted as indicating that there is a systemic “senescence signal” that coordinates autumn senescence within a tree. However, the nonuniform senescence behavior in our grafted trees showed unequivocally that, at least in *Populus*, senescence timing is strictly dependent on the genetic background of the branch. Second, in contrast to the effect on bud set, the effect of GI on autumn-induced senescence is not mediated by FT, whose expression level did not influence autumn senescence, even under field conditions. FT is probably involved indirectly, as growth cessation and/or bud set in some way predisposes the tree to sense the elusive “autumn signal.” This is consistent with our previous findings that the light signal that triggers induction of autumn senescence in aspen is not daylength per se (Michelson et al., 2017), which is believed to act through FT (Ding et al., 2018). Instead, another light signal seems to be sensed and transduced through GI to trigger autumn leaf senescence.

Our results do, however, also give information about leaf senescence not directly triggered by an “autumn signal.” Reducing *GI* expression resulted in both reduced growth and aberrant leaf development, and made the leaves sensitive to photooxidative stress. Grafting onto a WT rootstock that could provide the mutant with mobile signals such as FT rescued growth but not the increased starch accumulation, increased C/N ratio of the leaves, and initiation of senescence in intervein regions in the leaf under LD^18^ ^h^ (Figures 4 and 8). Lowering *GI* expression in Arabidopsis leads to malfunctions in phloem transfer cells or stomata (Edwards et al., 2010; Ando et al., 2013), and our data suggest that the same is also happening in *Populus*, causing a reduction in photosynthesis in GI-RNAi (8-2) leaves and leading to the senescence phenotype. In general, C/N imbalance is often associated with leaf senescence (Wingler et al., 2006; Aoyama et al., 2014; Chen et al., 2015; Fataftah et al., 2018), but it is unclear whether the C/N imbalance that we noted in the leaves is a cause or a consequence of the changed photosynthetic performance. Stomatal malfunction may also cause an array of effects, and photosynthesis gas exchange could be compromised, which would hamper photosynthesis, lead to downregulation of photoinhibition, and so on. The relation between *GI* expression and stress is, however, complex. For example, a knockout *gi* mutant is more resistant to herbicides, salt, or external H_2_O_2_ (Cao et al., 2006; Kim et al., 2013; Cha et al., 2019) and downregulation of *GI*-like genes confers salt stress tolerance on *P. alba* × *glandulosa* (Ke et al., 2017). However, *GI* mRNA levels increase severalfold in cold-treated Arabidopsis plants (Fowler and Thomashow, 2002), and the knockout mutant is more sensitive to low temperature (Cao et al., 2005). More studies are needed to find out how exactly *GI* expression relates to stress in different plant species, developmental stages, and light intensities, and whether this response is dependent on or independent of stomatal conductance and carbohydrate status.

If not FT, what are the molecular players downstream of GI that regulate autumn leaf senescence? Furthermore, why do the regions close to veins sometimes senesce earlier, sometimes later in GI-RNAi leaves? Several reports suggest that the photoperiodic components are expressed mainly in stomata and vascular tissues (An et al., 2004; Imaizumi et al., 2005; Para et al., 2007; Adrian et al., 2010; Edwards et al., 2010; Kinoshita et al., 2011; Ando et al., 2013). Control of nutrient and signal flow by stomatal function and/or vein function (including xylem unloading or phloem loading) could potentially explain how the light signaling/circadian clock controls leaf physiology. A possible connection between these pathways could be ethylene or reactive oxygen species (ROS). Haydon et al. (2017) demonstrated that ethylene shortens the circadian period in Arabidopsis, in a manner conditional on the effects of sucrose and requiring GI. In addition, PHY interacting factors, targeted by the GI pathway (Nohales et al., 2019), are involved in both ethylene biosynthesis and signaling pathways during dark-induced leaf senescence (Sakuraba et al., 2014; Song et al., 2014).

To conclude, reduced expression of *GI* in *Populus* leaves resulted in dramatic changes in leaf physiology causing alterations in, for example, leaf shape, C/N ratio, photosynthesis, and, most notably, leaf senescence through two different pathways, which appear to be independent of the expression of *FT* and bud set.

## Materials and methods

### Plant material and growth conditions

Hybrid aspen *P.* *tremula L.* × *Populus tremuloides* Michx, clone T89 (WT), FT-RNAi, *FT*-ox, GI-RNAi lines (8-2 and 1-1a), and *GI*-ox, which were characterized in Böhlenius et al. (2006) and Ding et al. (2018) were obtained from the tissue culture facility (Umeå Plant Science Centre) and potted in soil in 3-L pots. The saplings were grown in the greenhouse under the following conditions: LDs (18/6 h day/night), temperature (20/15°C day/night), light intensity (200 µmol m^−2^. s^−1^) and 60% relative air humidity. The trees were fertilized weekly with 100 mL of diluted fertilizers (1:100; V:V) (SW Horto company, Hammenhög, Sweden).

### Grafting experiments

Plants were grown in soil in the greenhouse, and then scions were grafted onto root stocks; each graft was covered with a plastic bag until the graft was established. Different tree development stages, with 5, 10, and 15 leaves, were also tested. The grafted trees were initially grown under LD conditions before transfer to different growth conditions.

### Chlorophyll, anthocyanin, and flavonol indices

CCI of leaves (mean of five leaves) was determined for at least four independent trees from each genotype using a chlorophyll meter (CCM 200 plus, Opti-Sciences). The anthocyanin and flavonol indices were measured using Dualex Scientific+ (Force-A). For each biological replicates, five leaves were measured and their values averaged.

### Simulating autumn conditions

Nongrafted and grafted trees were transferred from LD to SD (14/10 h; 20/15°C day/night) (Figure 2A). After 2 weeks, the trees were subjected to cold night conditions (18/5°C day/night), then after a further 2 weeks the photoperiod was altered to 12/12-h (day/night). Fertilization was stopped after 3 weeks in SD conditions coinciding with the decrease in growth and the start of the dormancy stage.

### Second growth cycle

The trees that had experienced the simulation of autumn conditions were subjected to a temperature of 5°C and a photoperiod of 8/16 h (day/night) for 2 months to break dormancy. The trees were re-potted in 10-L pots and flushed again under LD conditions. A similar experiment setup to that mentioned in the previous section was used to study leaf senescence under simulated autumn conditions for these trees.

### Starch staining and leaf weight measurement

For starch staining, leaves were bleached with 80% EtOH at 80°C for 10–20 min until the chlorophyll was completely removed from the leaves. The bleached leaves were stained with iodine solution containing 0.7% KI (W/V) and 0.3% I (W/V) for 3 min. The leaves were rinsed with water and photographed.

For weight measurements, several discs were obtained in replicate from 10 leaves. Leaf fresh weight and leaf area were measured, then the leaves were dried at 70°C for 48 h to measure the dry weight.

### Gas exchange and chlorophyll fluorescence measurement

The A_n_, g_s_, and Ci of the leaves were measured using a portable CO_2_ infrared gas analyzer (LI-6400XT, LI-COR Environmental, USA), equipped with a chamber that controlled irradiance (1,000 µmol photons m^−2^ s^−1^), temperature (20°C), CO_2_ concentration (400 µmol mol^−1^), and flow rate (250 cm^3^ min^−1^). The measurements were tested at zeitgeber (ZT) 3, 6, and 9 h after the light went on. The differences between the WT and GI-RNAi leaves were reproducible at the different ZT. Accordingly, data for ZT9 are presented here.

For chlorophyll fluorescence imaging of the leaves, we used a SPEEDZEN imaging system, and recorded an induction curve (after 30 min of dark incubation under ambient O_2_ and CO_2_ conditions) with 2,600 µmol actinic light for 3.5 min with a 6,000 µmol saturating pulse at 30-s intervals to attain maximum NPQ levels without causing artificial damage during measurements, followed by 3.5 min of dark recovery; this experimental protocol was found to be adequate for the aspen leaves, as fluorescence had saturated after the 7th flash, and had returned to low levels after the 14th. NPQ formed by short exposure with high light is normally known as fast NPQ (pH-dependent), whereas the residual fluorescence after dark recovery is usually known as the slow component of NPQ (Zeaxanthin/qI).

### RNA extraction and quantitative polymerase chain reaction (qPCR)

Mature poplar leaves were harvested at zeitgeber 9 or 17, immediately frozen in liquid N_2_ and ground to a fine powder with a mortar and pestle. About 100 mg powder was used for RNA extraction with CTAB extraction buffer (Chang et al., 1993; 2% CTAB, 100 mM Tris–HCl (pH 8.0), 25 mM EDTA, 2 M NaCl, 2% PVP). The samples were incubated at 65°C for 2 min and extracted twice with an equal volume of chloroform-isoamylalcohol (24:1). Nucleic acids were precipitated at −20°C for 3 h with one-quarter volume 10 M LiCl. Precipitate was collected by centrifugation (13,000 rpm, 4°C, 20 min) and purified and DNase treated with an RNeasy kit according to the manufacturer’s instructions (RNeasy mini kit (reference number 74104); Qiagen GmbH, Hilden, Germany). RNA integrity was confirmed by agarose gel electrophoresis. About 1,000 ng RNA was used for cDNA synthesis with an iScript cDNA Synthesis Kit (BioRad, Hercules, CA, USA). The cDNA was diluted 50 times for downstream applications. Quantitative real-time PCR was run on a LightCycler 480 with SYBR Green I Master (Roche, Basel, Switzerland). All kits and machines were used according to the manufacturer’s instructions. The reaction protocol started with 5 min pre-incubation at 95°C, followed by 50 cycles of amplification consisting of 10-s denaturation at 95°C, 15 s annealing at 60°C, and 20-s elongation at 72°C. For the acquisition of a melting curve, fluorescence was measured during the step-wise increase in temperature from 65°C to 97°C. Relative expression levels were obtained using the 2^−^^ΔΔCq^ method (Livak and Schmittgen, 2001). GeNorm identified UBQ and 18S as most stable reference genes. All primers used had an efficiency of >1.8 and their correct products were confirmed by sequencing. The primers sequence were: *GI* (forward: 5′-CAATGAAACCCGCTTCTAAACTCA-3′; reverse: 5′-AGCTTGCCAGTTGATGACATCTG-3′), *FT2* (forward: 5′-AGCCCA AGGCCTACAGCAGGAA-3′; reverse: 5′-GGGAATCTTTCTCTCATGAT-3′), *UBQ* (forward: 5′-GTTGATTTTTGCTGGGAAGC-3′; reverse: 5′-GATCTTGGCCTTCACGTTGT-3′), and *18S* (forward: 5′-TCAACTTTCGATGGTAGGATAGAG-3′; reverse: 5′-CCGTGTCAGGATTGGGTAATTT-3′).

### C/N ratio measurement

Dried leaves were pooled and ground with a mortar and pestle. Dry mass was defined after oven drying at 70°C for 24 h. C and N concentrations (mass based) of the samples (5-mg dry weight) were determined with an elemental analyzer (Flash EA 2000, Thermo Fisher Scientific, Bremen, Germany) using four biological replicates. The C and N of the dried sample material were converted to CO_2_ and N_2_ by combustion. The results were corrected for drift and sample size effect (nonlinearity). Working standards were wheat and maize flours calibrated against reference standards. For ωN, the standards were atropine, cellulose, and NIST 1515 apple leaves (Merck company). For ωC, they were cyclohexanone, nicotinamide, and sucrose.

### Outdoor experiment

For FT, the hybrid aspen plants (T89), FT-RNAi, and *FT1*-ox lines that were characterized by Böhlenius et al. (2006), were obtained from the tissue culture facility and every tree was potted in 3 L of soil. The plants were kept in a greenhouse and subjected to a light period 18 h at a light intensity of 200 µmol m^−2^. s^−1^. The temperature was 20/15°C (day/night). Fifty days after recovery, the plants were transferred to natural conditions outside the greenhouse (on August 22, 2019).

For GI, two groups of trees were used in this experiment. The first group consisted of trees that were simply grafted onto WT rootstock as described in Figure 1B. The second group was grafted using Y method grafting as illustrated in Figure1B. The trees completed the first growth cycle and after the dormancy break, the WT buds were removed from the first group of trees. All trees were re-potted in 10-L pots and flushed in the greenhouse in June 2020. At the beginning of July, the trees were moved out of the greenhouse (Umeå, Sweden) to undergo autumnal senescence, exposed to natural conditions such as light and temperature.

### Field experiment

This experiment was a part of a larger trial of genetically modified aspens. All genotypes were obtained from the tissue culture facility (Umeå Plant Science Centre) and potted in the greenhouse. The trees were then planted in at a field site at Våxtorp, southern Sweden, in 2014. Height and chlorophyll content were measured for 2-year-old trees.

### Statistical analyses

The multiple ways analysis of variance analysis was performed using the Info- Stat/Student program (http://www.infostat.com.ar/index.php?mod=page&id=37) and Fisher’s Least Significant Difference values were calculated for statistical analyses. In addition, *t* tests were used for two-group statistical analyses. A difference at *P* < 0.05 was considered as significant.

### Accession numbers

Sequence data from this article can be found in the GenBank/EMBL data libraries under accession numbers: *FT1*: Potra001726g14043; *FT2*: Potra001246g10694; *GI*: Potra001576g13038; *GIL*: Potra002370g18052; *UBQ*: Potra000756g05965; *18S*: AY652861.1

## Supplemental data 

The following materials are available in the online version of this article.

**Supplemental Figure S1.** The growth phenotype, anthocyanins, and flavanols of grafted GI-RNAi scions in the second growth cycle.

**Supplemental Figure S2.** The expression of *GI* in WT and different GI-RNAi lines scions grafted on WT rootstock under LD^18^ ^h^ conditions.

**Supplemental Figure S3.** Gas exchange parameters of different *GI* expression genotypes as scions grafted on a WT rootstock.

**Supplemental Figure S4.** Grafting WT on top of grafted GI-RNAi (line 8-2) (graft-on-graft).

**Supplemental Figure S5.** Gas exchange parameters of graft-on-graft trees are shown in Supplemental Figure S4.

**Supplemental Figure S6.** Senescence phenotypes of GI-RNAi (line 1-1a) and *GI*-ox under SD and cold night conditions.

**Supplemental Figure S7.** Time course of photosynthetic response of WT and GI-RNAi (line 8-2) leaves from either exposed or shaded parts of trees.

**Supplemental Figure S8.** Senescence phenotype of GI-RNAi (line 8-2) in outdoor conditions.

**Supplemental Figure S9.** Changes in *FT* expression had no effect on autumn senescence.

## Supplementary Material

kiab439_Supplementary_Data

## Notes

N.F. and S.J. conceived the study, S.J. and O.N. supervised the experiments, and N.F. designed and performed most of them and analyzed the data. P.B. was responsible for the analysis of SPEEDZEN imaging data, D.A. and B.Z. for studies of gene expression. J.L. assisted in integrating the results and P.K.I. analyzed field experiment data. N.F. and S.J wrote the article and all authors discussed and commented on it. S.J. is the author responsible for contact and communication.

The author responsible for distribution of materials integral to the findings presented in this article in accordance with the policy described in the Instructions for Authors (https://academic.oup.com/plphys/pages/general-instructions) is Stefan Jansson (Stefan.jansson@umu.se).
