# Supplementary material for: GIGANTEA influences leaf senescence in trees in two different ways
Source: Plant Physiol. 2021 Sep 23;187(4):2435–50. doi: 10.1093/plphys/kiab439 (PMC8644469; doi:10.1093/plphys/kiab439)
Supplement: kiab439_Supplementary_Data [file kiab439_supplementary_data.pdf]

Supplementary Figures

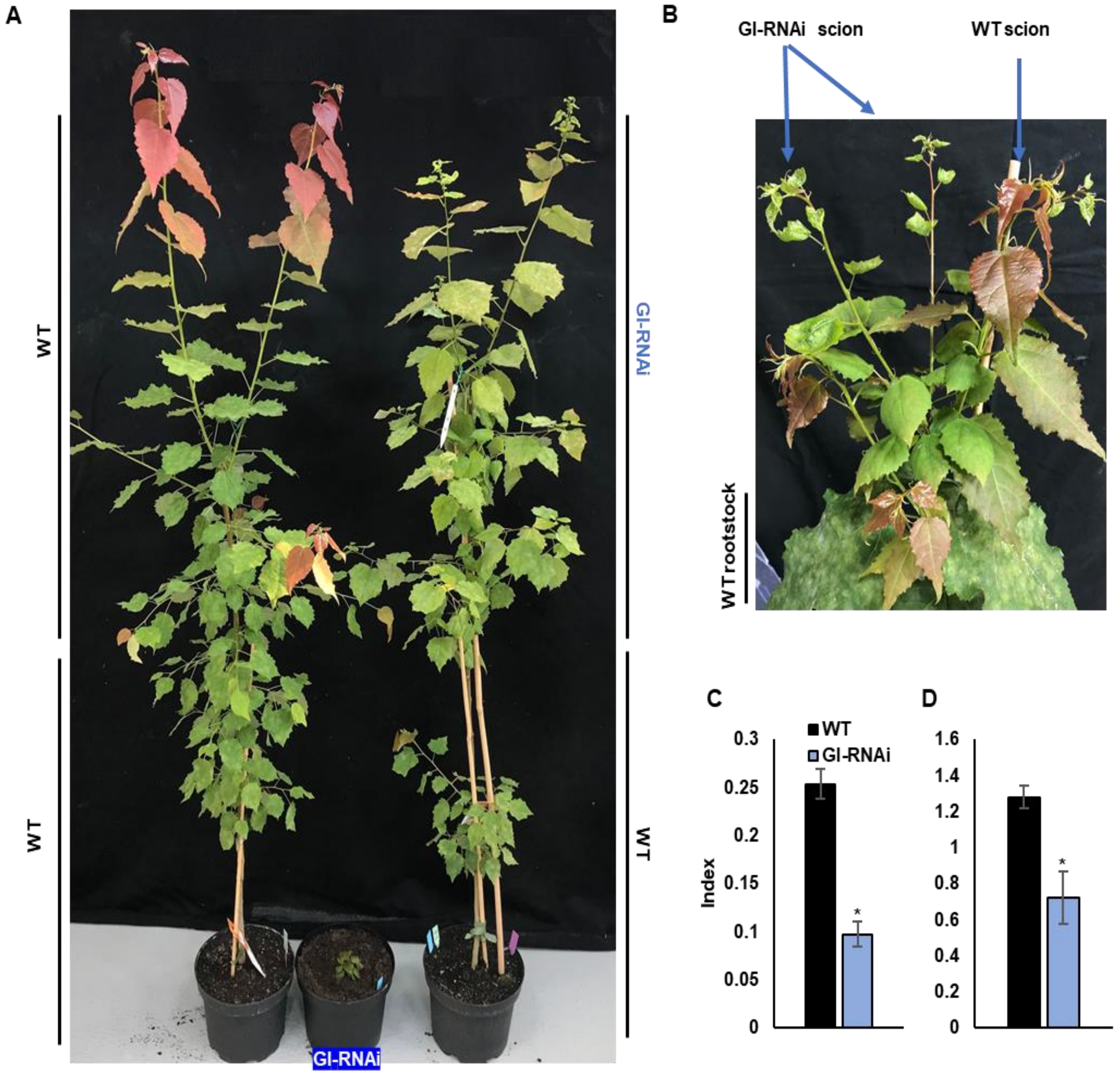

Supplemental Figure S1. The growth phenotype, anthocyanins, and flavanols of grafted GI-RNAi scions in the second growth cycle. A, growth of WT and GI-RNAi (line 8-2) scions grafted on WT rootstock in the second growth cycle after dormancy. B, WT and GI-RNAi scion grafted on WT rootstock using "Y" grafting as illustrated in Figure 1B. C-D, respectively the anthocyanin and flavanol indices for the growing leaves after bud flushing; The bars are the average of three biological replicates ±SD; the asterisk represents significant difference using a t-test; ($P < 0.05$). The trees were grown under LD^{18h} conditions.

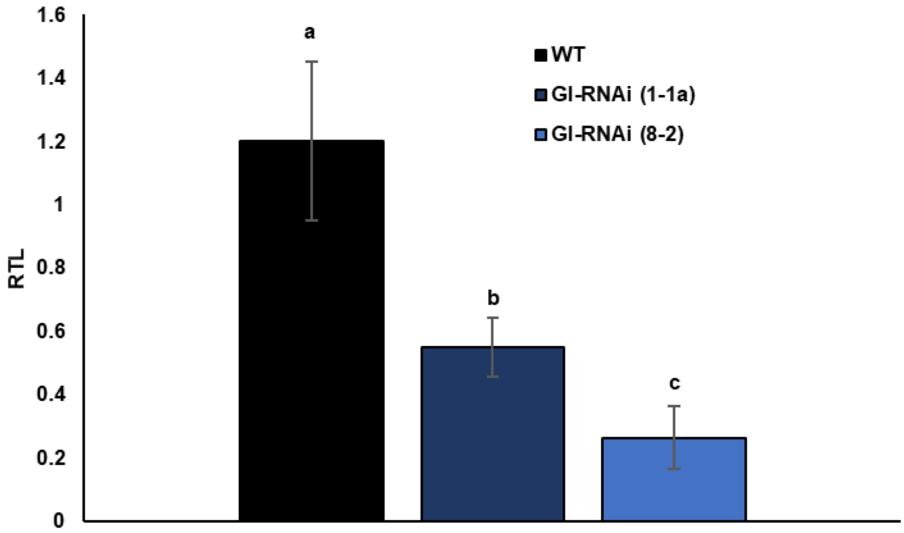

Supplemental Figure S2. The expression of *GI* in WT and different GI-RNAi lines scions grafted on WT rootstock under LD^{18h} conditions; RTL: relative transcription level; bar is the average of 5 biological replicates ±SD. Different letters represent significant differences using ANOVA analysis; $P < 0.05$.

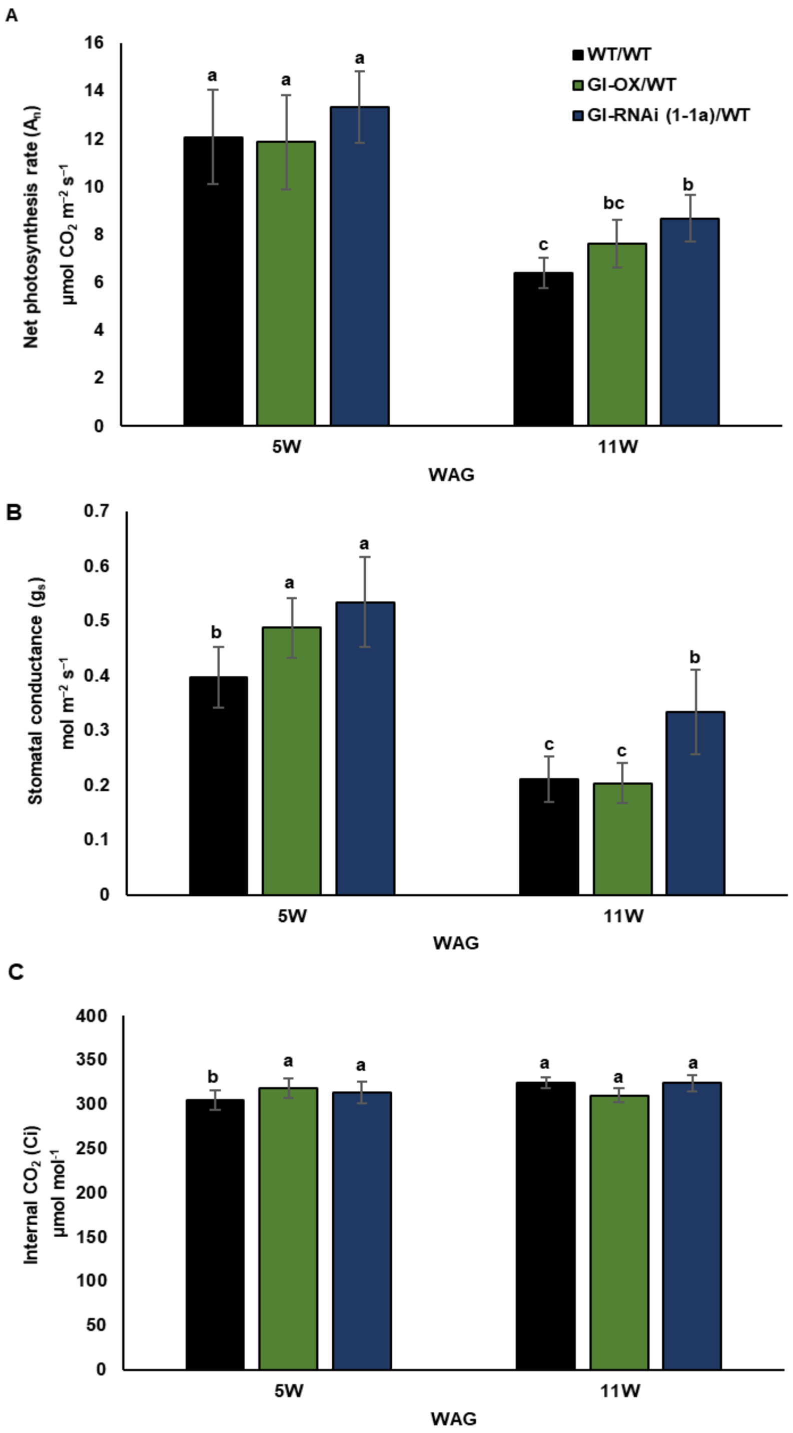

Supplemental Figure S3. Gas exchange parameters of different GI expression genotypes as scions grafted on a WT rootstock. A, maximum net CO_2 assimilation rate (A_n). B, stomatal conductance (g_s). C, internal CO_2 (Ci) of scion after grafting. WAG: week after grafting; the trees were moved to simulated autumn conditions at 8 WAG (see Figure 2A for the experimental setup). Bar is the average of 4 tree values ±SD. Different letters represent significant differences using ANOVA analysis; $P < 0.05$.

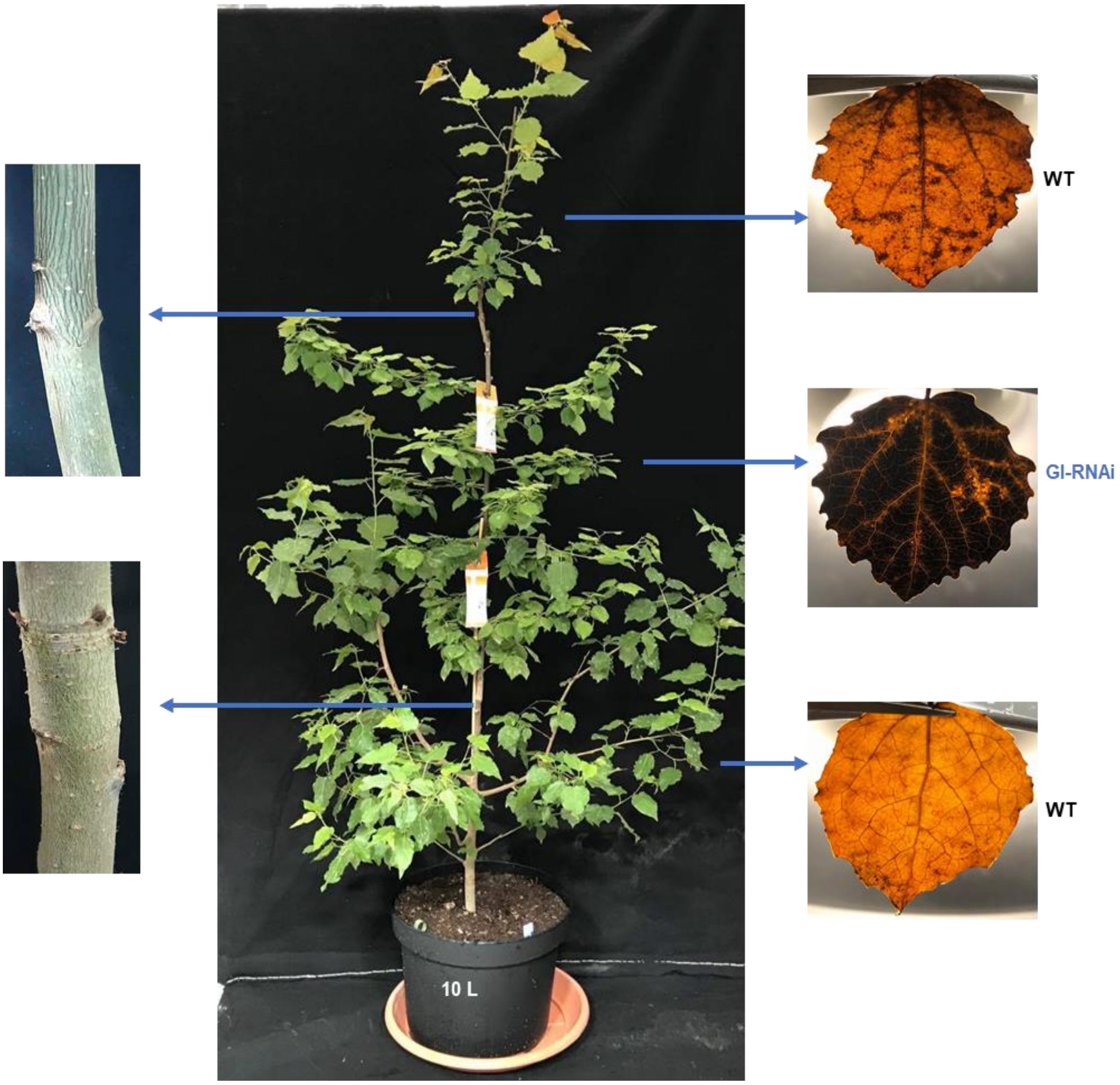

Supplemental Figure S4. Grafting WT on top of grafted GI-RNAi (line 8-2) (graft-on-graft). The tree undergoes dormancy and is shown here growing after bud flush in LD^{18h}. The left-hand pictures show stem diameter in the grafting regions. The right-hand pictures show the starch content in the leaves. Four trees were grafted (WT-on GI-RNAi-on WT), and three self-grafted WT were used as control.

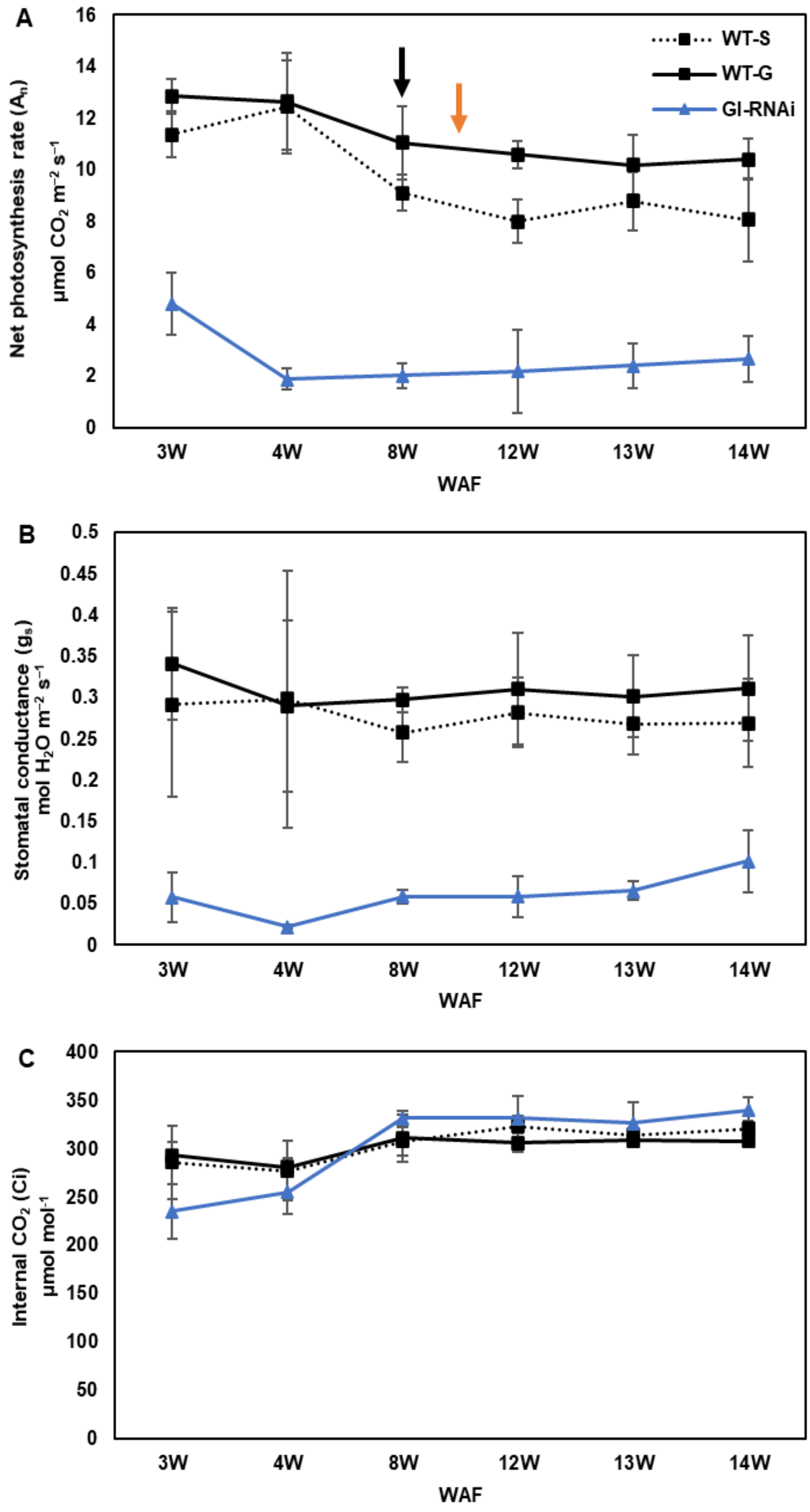

Supplemental Figure S5. Gas exchange parameters of graft-on-graft trees shown in Fig.S4. A-C, A_n, g_s, and Ci respectively; WT-S: self grafted WT. WT-G: WT scion grafted on GI-RNAi (line 8-2); WAF: weeks after flushing. The black arrow indicates when the trees were subjected to a short day, and the orange arrow shows when they were subjected to a cold night. Bar is the average of 4 tree values ±SD.

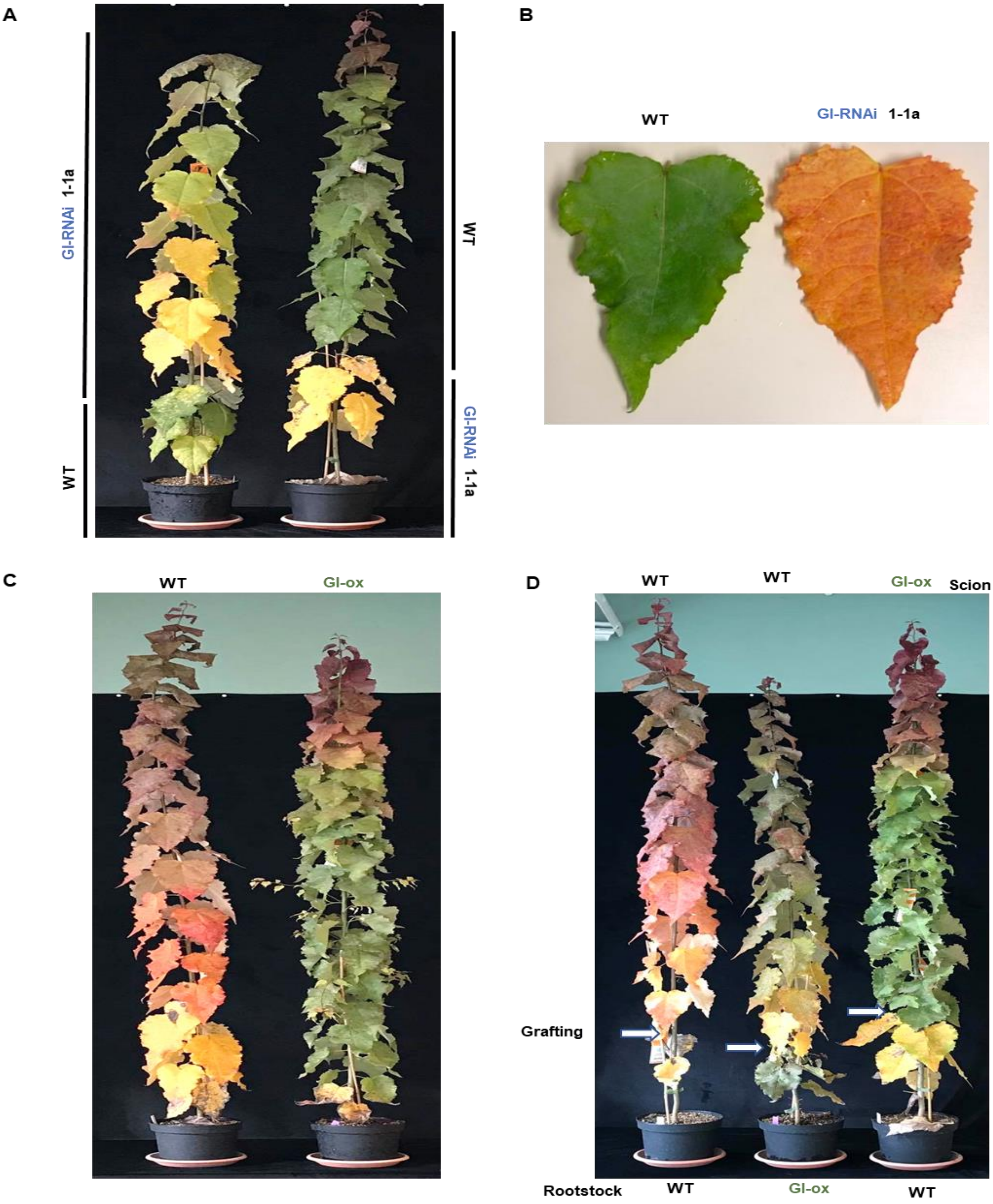

Supplemental Figure S6. Senescence phenotypes of GI-RNAi (line 1-1a) and GI-ox under SD and cold night conditions. A, senescence phenotype of GI-RNAi (line 1-1a) scions and rootstock under simulated autumn conditions; the photo was taken fifteen weeks after grafting (WAG). B, picture shows the uniform senescence phenotype of line 1-1a. C, senescence phenotype of ungrafted WT and GI-ox under simulated autumn conditions; the photo was taken twenty-three weeks after potting. D, senescence phenotype of grafted WT and GI-ox under simulated autumn conditions; the photo was taken twenty WAG. The reddish topmost leaves of the GI-ox tree are growing leaves which they are typically reddish in *Populus* trees.

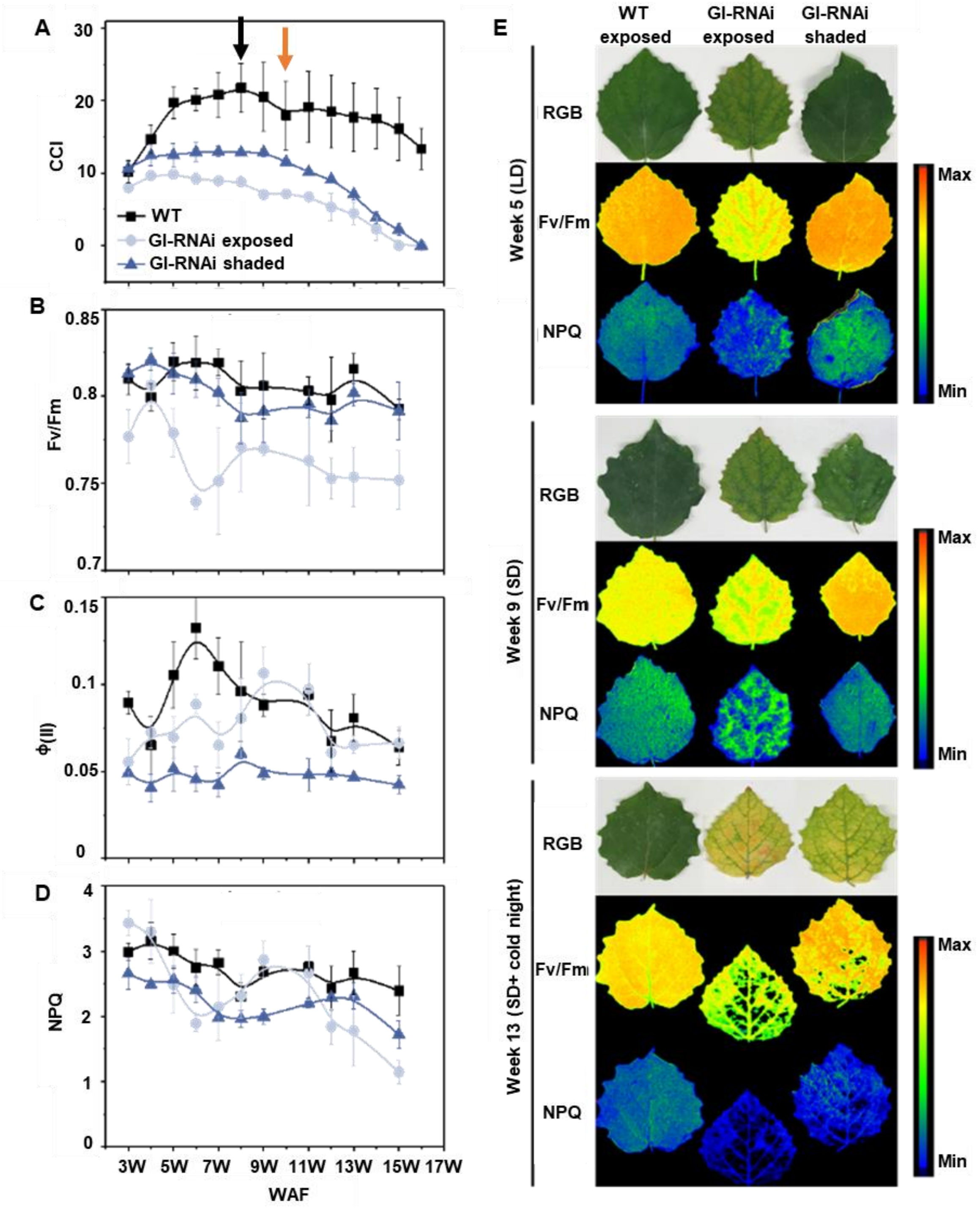

Supplemental Figure S7. Time course of photosynthetic response of WT and GI-RNAi (line 8-2) leaves from either exposed or shaded parts of trees in LD^{18h} (weeks 3-7), SD (black arrow, weeks 8-11) and SD + cold night (orange arrow, weeks 12-16) conditions. A, chlorophyll content index (CCI) was measured throughout the experiment. B, maximum quantum yield of PSII (Fv/Fm). C, effective quantum yield of PSII [Φ(II)] and D, non-photochemical energy dissipation (NPQ) measured with saturating pulse under 1000 µmole constant actinic light. E, Visible differences in fluorescent images showing Fv/Fm and NPQ from LD (Week 5), SD (Week 8) and SD + cold night (Week 13) conditions. Bar is the average of three biological replicates values ±SD.

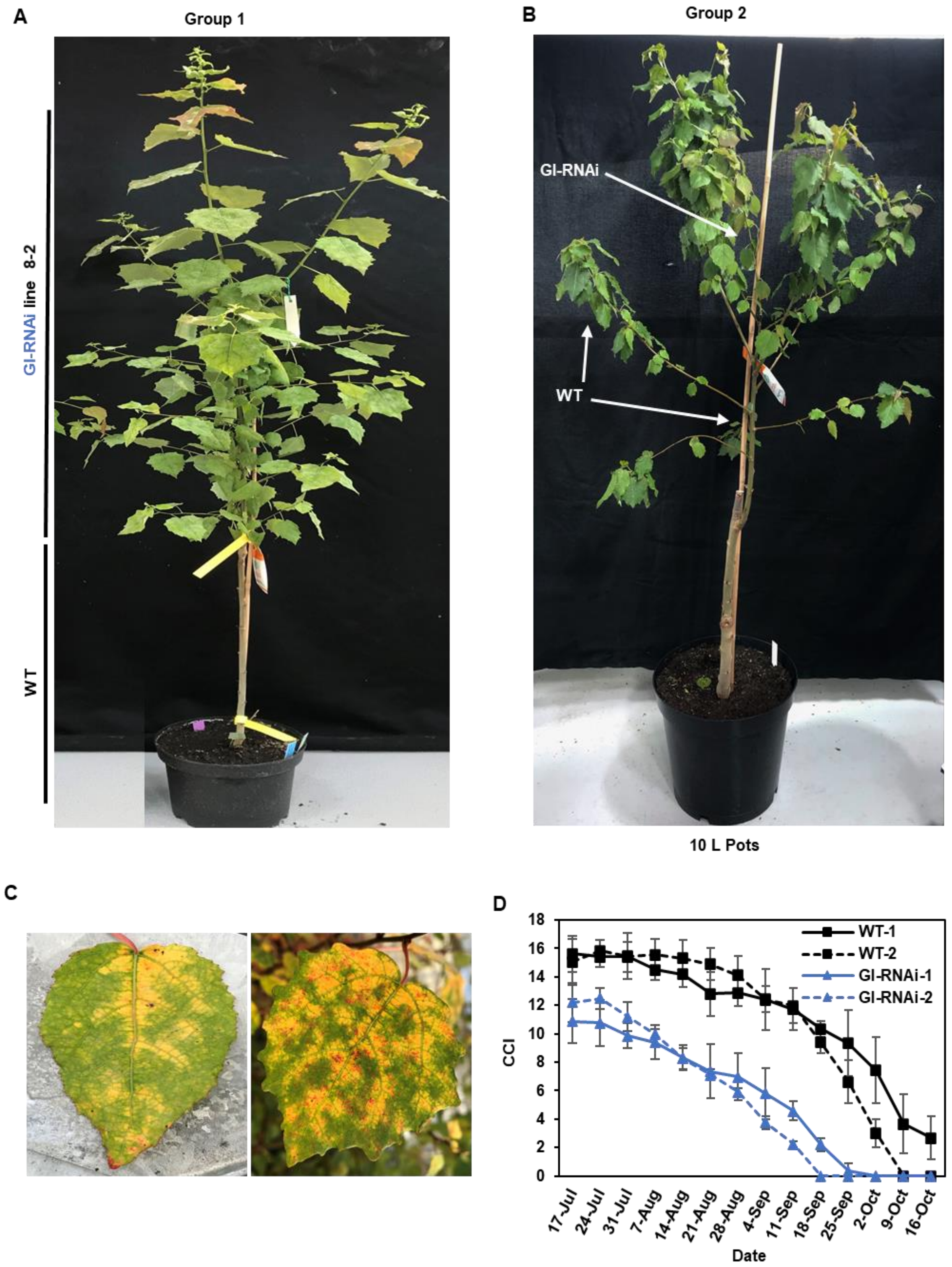

Supplemental Figure S8. Senescence phenotype of GI-RNAi (line 8-2) in outdoor conditions. A, normally grafted WT and GI-RNAi (line 8-2). B, GI-RNAi and WT scions grafted on WT rootstock (Y grafting). C, the spatial distribution of leaf senescence in GI-RNAi under outdoor conditions; September 2020. D, CCI of the two groups in outdoor conditions in Umeå; year 2020; each data point is the average of 3-4 tree values ±SD.

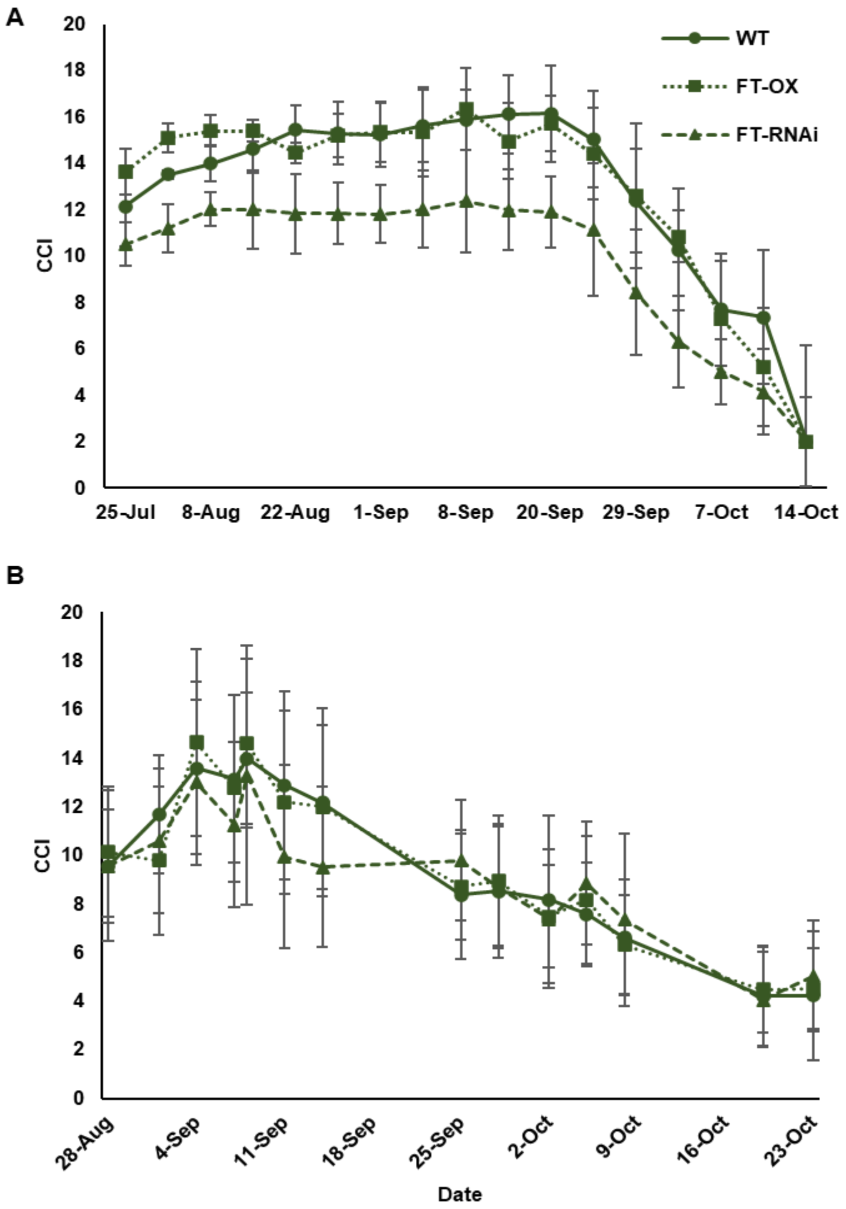

Supplemental Figure S9. Changes in *FT* expression had no effect on autumn senescence. A, chlorophyll content index (CCI) of WT, *FT*-ox, and FT-RNAi trees in their first growing cycle in the outdoor experiment in Umeå in year 2019; values are the average for six trees ±SD. B, CCI of WT, *FT*-ox, and FT-RNAi two-year-old trees in the field site in southern Sweden; values are averages for nine trees ±SD.
